# Multimodal workflows optimally predict response to repetitive transcranial magnetic stimulation in patients with schizophrenia: a multisite machine learning analysis

**DOI:** 10.1038/s41398-024-02903-1

**Published:** 2024-04-25

**Authors:** Mark Sen Dong, Jaroslav Rokicki, Dominic Dwyer, Sergi Papiol, Fabian Streit, Marcella Rietschel, Thomas Wobrock, Bertram Müller-Myhsok, Peter Falkai, Lars Tjelta Westlye, Ole A. Andreassen, Lena Palaniyappan, Thomas Schneider-Axmann, Alkomiet Hasan, Emanuel Schwarz, Nikolaos Koutsouleris

**Affiliations:** 1 Department of Psychiatry and Psychotherapy, University Hospital, Ludwig-Maximilian-University of Munich, Munich, Germany; 2 Max Planck Institute of Psychiatry, Munich, Germany; 3 Centre of Research and Education in Forensic Psychiatry, Oslo Univerisity Hospital, Oslo, Norway; 4 The University of Melbourne, Melbourne, VIC Australia; 5 Institute of Psychiatric Phenomics and Genomics (IPPG), University Hospital, LMU Munich, Munich, Germany; 6 Department for Psychiatry and Psychotherapy, Central Institute of Mental Health, Medical Faculty Mannheim, Heidelberg University, Mannheim, Germany; 7 Hector Institute for Artificial Intelligence in Psychiatry, Central Institute of Mental Health, Medical Faculty Mannheim, Heidelberg University, Mannheim, Germany; 8 Centre for Mental Health, Darmstadt-Dieburg District Clinic, Gross-Umstadt, Germany; 9 Partner site Munich-Augsburg, DZPG (German Centre for Mental Health), Munich / Augsburg, Germany; 10 Centre for Precision Psychiatry, University of Oslo, Oslo, Norway; 11 Division of Mental Health and Addiction, Oslo University Hospital, Oslo, Norway; 12 Douglas Mental Health University Institute, Department of Psychiatry, McGill University, Montreal, QC Canada; 13 Robarts Research Institute, Western University, London Ontario, Canada; 14 Department of Psychiatry, Psychotherapy and Psychosomatics, Medical Faculty, University of Augsburg, Augsburg, Germany; 15 Institute of Psychiatry, Psychology and Neuroscience, King’s College London, London, UK

**Keywords:** Predictive markers, Schizophrenia

## Abstract

The response variability to repetitive transcranial magnetic stimulation (rTMS) challenges the effective use of this treatment option in patients with schizophrenia. This variability may be deciphered by leveraging predictive information in structural MRI, clinical, sociodemographic, and genetic data using artificial intelligence. We developed and cross-validated rTMS response prediction models in patients with schizophrenia drawn from the multisite RESIS trial. The models incorporated pre-treatment sMRI, clinical, sociodemographic, and polygenic risk score (PRS) data. Patients were randomly assigned to receive active (*N* = 45) or sham (*N* = 47) rTMS treatment. The prediction target was individual response, defined as ≥20% reduction in pre-treatment negative symptom sum scores of the Positive and Negative Syndrome Scale. Our multimodal sequential prediction workflow achieved a balanced accuracy (BAC) of 94% (non-responders: 92%, responders: 95%) in the active-treated group and 50% in the sham-treated group. The clinical, clinical + PRS, and sMRI-based classifiers yielded BACs of 65%, 76%, and 80%, respectively. Apparent sadness, inability to feel, educational attainment PRS, and unemployment were most predictive of non-response in the clinical + PRS model, while grey matter density reductions in the default mode, limbic networks, and the cerebellum were most predictive in the sMRI model. Our sequential modelling approach provided superior predictive performance while minimising the diagnostic burden in the clinical setting. Predictive patterns suggest that rTMS responders may have higher levels of brain grey matter in the default mode and salience networks which increases their likelihood of profiting from plasticity-inducing brain stimulation methods, such as rTMS. The future clinical implementation of our models requires findings to be replicated at the international scale using stratified clinical trial designs.

## Introduction

Repetitive transcranial magnetic stimulation (rTMS) provides a non-invasive treatment option capable of inducing long-term excitability and plasticity changes at the neural-systems level across various neuropsychiatric disorders. rTMS has been most promising in the treatment of depression with overall milder adverse effects [1]. In other neurological or neuropsychiatric disorders such as stroke [2], Alzheimer’s disease [3], Parkinson Disease [4] and schizophrenia [5], rTMS has also shown to be effective. Specifically, a small number of investigations using rTMS as an alternative option to treat negative symptoms in schizophrenia have emerged over the recent years because these disabling symptoms do not respond adequately to antipsychotic or psychosocial treatments [6, 7]. However, rTMS treatment outcomes are observed to have large inter-individual variability. This heterogeneity may result from genetic [8], neuroanatomical [9], neurofunctional [10], connectivity-based [11], and sociodemographic [12] factors. So far, no study has analysed this multi-dimensional heterogeneity to develop individualised predictors of rTMS treatment outcomes, except for depression [13].

Treatment outcome prediction in schizophrenia has developed into an important area of precision psychiatry research [14, 15]. The emergence of machine learning and AI methodologies has provided researchers the means to create prediction models utilising multivariate and multimodal data. We previously predicted functional outcomes of first episode psychosis using psychosocial and symptoms variables and validated the model on an unseen sample of 108 patients with a balanced accuracy of 71.7% [16]. Leighton et al. successfully predicted 1-year remission and recovery outcomes to medication treatment in first psychosis and validated their findings in two independent samples using baseline clinical and demographic variables [17]. Wang et al. predicted antipsychotic medication treatment outcomes in schizophrenia with MRI and polygenic risk scores [18].

Currently only one study used machine learning to predict rTMS treatment outcomes in schizophrenia [6]. In this previous work, we developed and cross-validated an rTMS treatment response classifier for patients with predominant negative-symptom schizophrenia based on structural Magnetic Resonance Imaging (sMRI) as a single predictive modality. However, we did not assess the potential added value of clinical, sociodemographic, and genetic information available for these patients. Based on previous evidence showing a superiority of multimodal predictive models over unimodal models [19, 20], we hypothesised that the predictive power of our original, sMRI-based predictive model could be improved by integrating clinical, sociodemographic, and genetic information with imaging data. To this end, we combined the sMRI predictor with newly trained models analysing clinical, sociodemographic, and genetic data into a multimodal prediction system. Secondly, previous work [20] showed that the strategic combination of multiple data domains following the principles of deferral learning [21] may lead to more efficient predictive systems. By performing only those examinations in each patient that conjointly minimise individual predictive uncertainty such systems could be more easily translated to clinical care, thus reducing data acquisition costs and diagnostic burden on the patients.

Therefore, we hypothesised that sequential prediction techniques increase the clinical adaptiveness of rTMS response prediction models compared to “data-hungry” approaches that require the presence of all data in every patient to be tested, while maintaining the higher performance of the latter predictive strategies. Hence, we trained and validated a sequential predictive model using all available data domains in the “Repetitive Transcranial Magnetic Stimulation for the Treatment of Negative Symptoms in Schizophrenia” (RESIS) trial database. Thirdly, we evaluated the correlations between the sMRI, clinical and PRS data to identify any potential cross-modality associations. By doing so, we aimed at a deeper understanding of the underlining patterns determining the inter-individual variability of patients’ responses to rTMS.

## Methods

### Study subjects and target definition

The RESIS study recruited patients with an ICD-10 diagnosis of schizophrenia across three academic clinical centres, who met the following criteria: Positive and Negative Syndrome Scale, negative subscale (PANSS-NS) > 20 points, 1 PANSS-NS item ≥4, no PANSS-NS reduction ≥10% in the 14 days before treatment start, and an illness duration of ≥1 year [7]. All patients provided written informed consent prior to study enrollment. The study was registered at https://clinicaltrials.gov (NCT00783120) and the study protocol [22] was approved by the institutional review boards of the three participating institutions (University of Goettingen, University of Duesseldorf, University of Regensburg).

From the Intention-To-Treat (ITT) population (*N* = 157), 96 patients had pre-treatment sMRI (active/sham rTMS: *N* = 45/47) and primary PANSS-NS outcome endpoints defined as follows [7, 23]: ∆PANSS–NS% = (PANSS–NS_T1_ − PANSS–NS_Baseline_) × 100/(PANSS–NS_Baseline_ – 7). PANSS-NS_Baseline_-7 was used as baseline value instead of PANSS-NS_Baseline_ as 7 was the lowest possible value for PANSS-NS_Baseline_ [24]. The patients were assigned response or non-response labels, where response was defined as ≥20% improvement between baseline and day 21 in PANSS-NS. These labels were used as targets for the machine learning analyses described below.

### Treatment and intervention

All patients in the ITT population were blinded to the intervention and were randomised either to 10 Hz active or sham rTMS applied to the left DLPFC according to the EEG-10–20 system (F3-electrode, 5 sessions/week during the 3-week period, 1000 stimuli/day, 50 stimuli/train) with 110% of the individual resting motor threshold (RMS). The difference between the active and sham treatment was that sham-treated patients had the stimulation coil tilted over one wing at an angle of 45 degrees. Rater-blinded clinical data were recorded before stimulation (baseline/T0) and after day 21 (T1), day 28 (T2), day 45 (T3) and day 105 (T4). In the ITT population no significant differences in the primary outcome, other clinical outcomes and cognition could be detected between active and sham rTMS [7].

### Clinical and sociodemographic data acquisition and pre-processing

Only baseline data were used to train and cross-validate classifiers. We included all available clinical and sociodemographic data at baseline, no manual pre-selection was made to minimise manual intervention in the machine learning pipeline. The features included 16 clinical features consisting of the Positive and Negative Syndrome Scale Positive Score (PANSS-PS), Negative Score (PANSS-NS), General Score (PANSS-GS), Sum of Calgary Depression Scale for Schizophrenia items score (CDSS), Clinical Global Impressions Sickness Severity Score (CGI-S1), Global Assessment of Functioning score (GAF) and 10 items from Montgomery-Åsberg Depression Rating Scale (MADRS); 4 comorbidity features (life-time history of Alcohol abuse, Alcohol addiction, Substance abuse, Substance addiction prior to study recruitment) and 5 socio-demographic features (Marital status, Employment status, Housing status, Education (years), Sum of education years from parents) (Supplementary C1, S1).

### Genetic data acquisition and PRS calculation

All patients, including both active and sham treatment groups, were genotyped on the Infinium PsychArray-24 BeadChip (Illumina, San Diego, CA, USA). Based on genetic ancestry components, we identified 15 patients out of the 45 patients from each of the active and sham groups as ancestral outliers which had to be removed from further analysis steps. As a result, only 30 patients from the active group and 30 from the sham group provided PRS data in the machine learning analyses (Supplementary C2). Schizophrenia PRS (PRS-SZ) and educational attainment PRS (PRS-EA) were calculated using the PRS-Continuous Shrinkage method (PRS-CS) [25]. SZ and EA genome-wide association studies were used as discovery samples [26, 27]. The PRS-CS method generated different scores based on different assumptions of polygenicity (6 φ values from 1e−1 to 1e−6). All 12 PRS features (6 PRS-SZ and 6 PRS-EA) were z-transformed and used in the subsequent machine learning analyses.

### sMRI imaging data acquisition and pre-processing

Structural MR images were obtained on two 3T systems (Siemens Trio) and one 1.5T system (Siemens Sonata) using T1-weighted sequences [9]. All images were quality-controlled, and 4 study participants had to be removed due to poor image quality. All sMRI images were processed using the r1207 version of the Computational Anatomy Toolbox for SPM (CAT12) [28]. The sMRI images of the remaining patients were processed through automated tissue segmentation and high-dimensional stereotactic registration with Diffeomorphic Anatomical Registration Through Exponentiated Lie algebra (DARTEL) [29]. The resulting grey matter density (GMD) images were registered to the MNI-152 template and smoothed with an 8 mm Gaussian kernel. Further details relating to image acquisition and preprocessing can be found in our previous work [6]. The GMD images were flattened into vectors consisting of 71276 voxels as input features in the machine learning analyses.

### Machine learning strategy

**Fig. 1:**
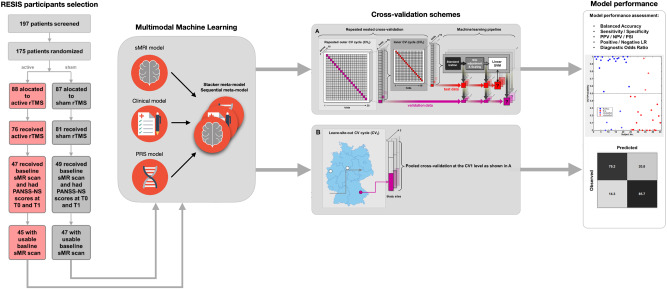
Schematic diagram of the main analysis design of our study. Boxes represent key analysis stages of our study, arrows represent the order of the analysis stages.

We generated machine learning models to predict rTMS treatment response with six different modality combinations using the open-source machine learning library NeuroMiner 1.1 [30] (Fig. 1). These modality combinations were (1) clinical and sociodemographic information (clinical model); (2) clinical, sociodemographic and PRS data (clinical + PRS model); (3) sMRI data; (4) stacked model combining the clinical and sMRI models (sMRI + clinical model); (5) stacked model combining all modalities (all-modalities model); and (6) an optimised sequential model combining all modalities (sequential model). These modality combinations were determined purely based on the availability of data domains in RESIS dataset.

The models were categorised into base, stacker, and sequential models. Base models did not follow a hierarchical stacking strategy: clinical, clinical + PRS and sMRI models. Stacker models employed a hierarchical meta-learning strategy that used the base models’ decision scores as input features to train a meta-classifier [20] that harnesses the predictive power of multiple modalities simultaneously with better explainability and flexibility. The sequential model combined different base and stacker models in a stepwise manner optimised for prediction performance and reduced the per-case examinations needed to achieve this performance, which is an innovative method to maximise the prediction accuracy by using multiple modalities while reducing the additional burden and cost for acquiring more data. The optimisation hyper-parameters included 7 candidate prognostic sequences, 5 upper and 5 lower case propagation percentile thresholds, resulting in 7 sequential models and a total of 175 hyper-parameter combinations (Supplementary 3.6).

We employed pooled repeated nested cross-validation (P-CV) with 10 permutations and 20 folds at the outer CV cycles, and 1 permutation with 19 folds at the inner CV cycles to achieve unbiased estimation of model generalisability to new patients. All models were trained using the linear kernel Support Vector Machine (SVM) algorithm. The optimisation metric was Balanced Accuracy: BAC = (Sensitivity + Specificity) ÷ 2. All SVM models generated from an inner CV cycle were combined into an ensemble classifier, which was then applied to the respective outer CV data to evaluate model performance. This process was repeated across all outer CV folds of the repeated nested CV design. For each patient in the outer CV fold, the obtained SVM decision scores were summed into one final prediction through majority voting. We employed three different preprocessing pipelines to cater for the different data domains of the six modality combinations (Supplementary 3.1. 3.2, 3.5). The pipelines were fully wrapped into each inner cycle of the CV structure to exclude any information leakage between training and test data.

We performed additional analyses to test model significance, generalisability, and therapeutic specificity. First, we conducted cross-over model validation by applying sham group data to active group models and vice versa. Then, we determined whether the observed prediction performances of the active and sham models were significant by training and cross-validating SVM models on *n* = 1000 random label permutations. Model significance was defined at *α* = 0.05 as *P* = _∑_^*n* = 1000^(BAC_(observed)_ ≤ BAC_(permuted)_) ÷ *n*. Next, we assessed the models’ generalisability by training models with leave-one-site-out cross-validation (LOSO-CV). This cross-validation scheme is a form of internal-external validation recommended for evaluating the generalisability of machine learning models in multi-centred studies as an alternative to external validation and can effectively evaluate overfitting [31]. Each of the three study sites was iteratively held-out for validation, while the remaining data entered the inner CV cycles. Consequently, the outer CV only had three folds and the training sample sizes for each fold were 28, 23, 39 respectively. Compared to the P-CV approach, the outer CV training sample sizes were reduced. The inner CV scheme was randomly pooled with 15 folds and 10 permutations. We observed that all LOSO-CV models showed lower prediction performances compared to the P-CV models in the active groups. To investigate whether the performance drop was due to residual site effects, or due to the lower training sample sizes caused by LOSO, we trained the three LOSO-CV base models on *n* = 1000 permutations of the patients’ site assignments. Additionally, we performed *Z*-test on all models trained on active-treated patients to assess whether the performance differences between the models were statistically significant (Supplementary 3.7, 3.8).

### Predictive pattern extraction

We used additional post hoc methods to extract the predictive patterns of the models. Specifically, for the sMRI model, we identified the reliability of the baseline GMD pattern using the Cross-Validation Ratio (CVR) method, mapped the significant regions onto the AAL brain atlas, and summarised the significant regions according to brain networks defined by the Yeo atlas using the open-source software MRIcroGL (Supplementary 4.3). For the clinical and PRS models, we used CVR, feature weights, Spearman coefficients, and sign-based consistency metrics to rank the features and identify the most predictive variables.

### Post hoc cross-modalities correlation analyses

We implemented a series of post hoc analyses to assess the correlation between clinical, PRS data and sMRI-based variables to find potential cross-modality patterns which could bridge the predictive patterns identified by the sMRI model and the clinical+PRS model in the active rTMS group. First, we corrected for covariate effects in all modalities following the same preprocessing pipeline used in the model development (Supplementary 3.1, 3.2). Then, we conducted univariate Pearson correlation analyses between each clinical and PRS feature used in our clinical + PRS model (Supplementary S15) and the GMD images organised in ROIs and brain networks (Supplementary C5).

### Post hoc predicted treatment effects analyses

We implemented a set of further post hoc analyses to investigate the relationship between the prediction results of our models and the precise PANSS-NS score reductions observed at different follow-ups after the patients received the treatment. These analyses included linear regression *R*² and *T*-test Cohen’s *d* calculations (Supplementary C6).

## Results

### Sample characteristics

Group level differences between the active and sham-treated groups are listed in Table 1. We did not find significant group level differences in basic sociodemographic variables, including sex (*p* = 0.315), site distribution (*p* = 0.886), right-handedness (*p* = 0.778), age (*p* = 0.418) and education (*p* = 0.830). Similarly, we did not find clinical baseline differences except for slightly higher PANSS-PS scores in the active rTMS groups (all PANSS-PS^Active^ = 14.4, PANSS-PS^Sham^ = 12.4, *p* = 0.012). This trend was similarly observed in PANSS-NS and GS, but not significant in both cases. Both treatment groups improved similarly over time (PANSS: all *F* ≥ 10.51, all *p* < =0.002; MADRS: *F* = 17.27, *p* < 0.001; GAF: *F* = 16.24, *p* < 0.001). Distributions of PANSS-NS responders and non-responders were equal in both rTMS treatment groups (active vs. sham rTMS responders/non-responders: 21/24 vs. 22/25; *χ*^2^ < 0.001, *p* = 0.989). Even though PANSS-PS scores were significantly higher in the active group at baseline (*t* = 2.565, *p* = 0.012), the significance was no longer observed at day 21 (*t* = 0.876, *p* = 0.383).

**Table 1:** Sociodemographic and clinical differences at baseline between Active and Sham rTMS treatment groups from RESIS dataset.

	Active rTMS	Sham rTMS	Active vs. Sham	
Sociodemographics	(*N* = 45)	(*N* = 47)	*χ*^2^	*p*
Sex (male:female)	39:6	37:10	1.01	0.315a
Site (Goettingen:Regensburg:Düsseldorf)	17:22:6	17:22:8	0.242	0.886a
Hand preference (Right:Left)	39:5	39:6	0.08	0.778a
Marital status (Married:Not married)	8:36	8:37	0.05	0.800a
Employment status (Working:Not working)	10:35	4:41	2.11	0.146a
Housing status (Live alone:Live with someone)	34:11	38:7	0.63	0.429a
Comorbidity				
Life-time history of alcohol abuse before study (Yes:No)	2:42	1:39	0.01	0.933a
Life-time history of alcohol addiction before study (Yes:No)	1:43	1:40	0.44	0.506a
Life-time history of substance abuse before study (Yes:No)	8:35	4:37	0.72	0.397a
Life-time history of substance addiction before study (Yes:No)	5:38	2:38	0.48	0.490a

Sociodemographics	Mean (SD)	Mean (SD)	*T*-stat	*p*
Age (years)	34 (9.9)	35.6 (9.4)	0.814	0.418b
Education (years)	11.4 (1.9)	11.3 (2.1)	0.215	0.830b
rTMS functional and anatomical parameters				
Left resting motor threshold (RMT)	46.7 (10.3)	48 (11.8)	0.519	0.605b
Scalp-to-cortex distance BA 9 (mm)	16.3 (2.2)	16.7 (1.9)	−0.951	0.344b
Scalp-to-cortex distance BA 46 (mm)	16.3 (2.4)	16.9 (2.5)	−1.211	0.299b
Severity of illness and treatment				
PANSS negative symptoms	26.3 (4.5)	25.9 (4.4)	0.428	0.669b
PANSS positive symptoms	14.4 (4.3)	12.4 (3.2)	2.565	0.012*b
PANSS general symptoms	42.4 (9.5)	38.7 (9.9)	1.766	0.081b
PANSS total	83.2 (14.2)	77.3 (14.8)	1.939	0.056b
Clinical global impressions: sickness severity score	4.6 (0.9)	4.7 (0.9)	−0.11	0.911b
Global assessment of functioning	52.1 (12.3)	52.4 (11.8)	0.115	0.908b
Antipsychotic dose (CPZ mg)	598.6 (451.1)	596.6 (494.5)	0.02	0.984b
Depression severity				
Calgary Depression Scale for Schizophrenia score	5.2 (3.4)	5.9 (3.5)	−0.83	0.411b
Montgomery–Åsberg Depression Rating Scale	14.5 (5.5)	13.8 (5.6)	0.558	0.579b

Sociodemographic and clinical differences were assessed using independent *t*-tests and chi-square tests. *p*a value obtained from chi-square test on independence. *p*b value obtained from independent *t*-test. All *p* values are FDR corrected using Benjamini/Hochberg method.

*PANSS* Positive and Negative Syndrome Scale, *CPZ* chlorpromazine equivalents, *BA* Brodmann area, *SD* standard deviation, *χ*^2^ chi-square test statistics, *T-stat* independent *T*-test statistics.

### Unimodal classifiers performances

**Fig. 2:**
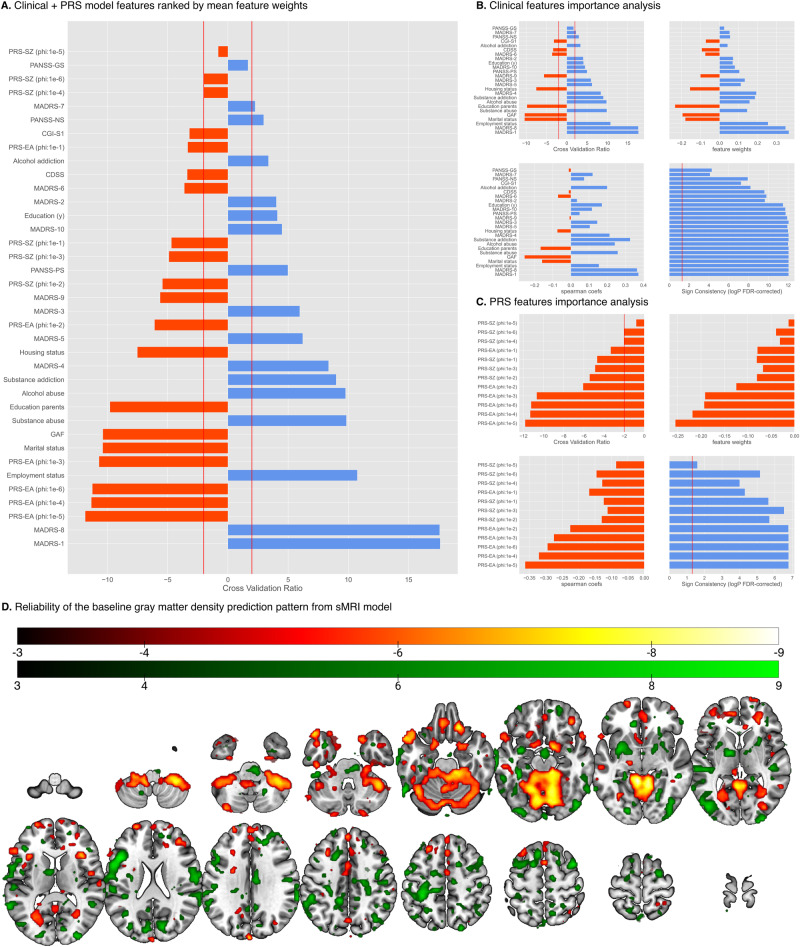
Feature importance and predictive patterns extracted from the Clinical+PRS model and sMRI model. **A** All features from Clinical + PRS model ranked by absolute CVR values in ascending order. The vertical red lines indicate CVR value at −2 and 2 which are equivalent to *p* = 0.05. **B** Predictive pattern analysis for the clinical and sociodemographic features used in the Clinical + PRS model, ranked by absolute CVR values in ascending order. CVR subplot vertical red lines: CVR equivalence to alpha level of 0.05 (|2.2|), Sign-based consistency subplot vertical red line: −log10 *p* equivalence to alpha level of 0.05 (1.3). **C** Predictive pattern analysis for the PRS features used in the Clinical + PRS model, ranked by absolute CVR values in ascending order. CVR subplot vertical red lines: CVR equivalence to alpha level of 0.05 (−2.2), Sign consistency subplot vertical red line: −log10 *p* equivalence to alpha level of 0.05 (1.3). All exact values can be found in Supplementary S15. **D** The reliability of the Grey Matter Density (GMD) pattern elements was measured in terms of a Cross-Validation Ratio (CVR) map (CVR = mean(**w**)/standard error(**w**)], where **w** are the weight vectors of the 5054 Support Vector Machine (SVM) models generated in the study’s repeated nested cross-validation setup). The CVR map was thresholded at CVR value ranges corresponding to an alpha level of 0.01 (CVR ≤ −3, CVR ≥ 3). Reliable areas of GMD increase in predicting responders to active rTMS are shaded in red colours, whereas areas of GMD increments predicting non-responders to active rTMS are painted in green. The open-source 3D rendering software MRIcroGL (C. Rohrden) available at https://www.nitrc.org/projects/mricrogl/ was used to overlay the CVR map on the MNI single-subject template.

All model performances can be found in Table 2. The clinical model achieved a BAC of 64.6% (sensitivity: 62.5%, specificity: 66.7%). The clinical + PRS model performed at a BAC of 75.9% (sensitivity: 70.8%, specificity: 81.0%), which was 11.3% higher than the performance of the clinical model (*p* = 0.009) (Supplementary S11). The top 10 most predictive features according to CVR included Apparent Sadness (MADRS-1), Inability to feel (MADRS-8), 4 PRS-EA scores (phi = 1e−5, 1e−4. 1e−6, 1e−3), employment status, marital status, GAF score and substance abuse (Fig. 2A–C). The retrained sMRI model with images processed using the CAT12 r1207 pipeline achieved a BAC of 80.1% (sensitivity: 79.2%, specificity: 81.0%). Compared to our previous work (BAC = 84.4%), our retrained sMRI model’s BAC was 4.3% lower, but not statistically significant different from the original sMRI model (*p* = 0.108).

**Table 2:** Validated predictive performances of Active and Sham rTMS response predictors.

ML model	*N*	TP	TN	FP	FN	BAC		AUROC	Sens	Spec	PPV	NPV	PSI	LR+	DOR	*p* value
PANSS-NS outcome predictor trained on Active rTMS patients																
Pooled repeated nested CV (P-CV)							BAC_Sham_									
Clinical	45	15	14	7	9	64.6	50.0	0.68	62.5	66.7	68.2	60.9	33.3	1.9	3.5	0.031*a
Clinical + PRS (early fusion)	45	18	15	6	6	75.9	50.0	0.77	70.8	81	81	70.8	51.8	3.7	13.8	0.001*a
sMRI	45	19	19	2	5	80.1	53.6	0.85	79.2	81	82.6	77.3	59.9	4.2	17.3	<0.001*a
sMRI + Clinical (stacker)	45	20	19	2	4	89	50.0	0.92	87.5	90.5	91.3	86.4	77.7	9.2	84.4	<0.001*a
All modalities (stacker)	45	20	20	1	4	89.3	50.0	0.96	83.3	95.2	95.2	83.3	78.6	17.5	306.2	<0.001*a
Sequential stacker	45	22	20	1	2	93.5	50.0	0.99	91.7	95.2	95.7	90.9	86.6	19.2	370.6	<0.001*a
Leave-one-site-out repeated nested CV (LOSO-CV)																
Clinical	45	7	14	7	17	47.9		0.49	29.2	66.7	50	45.2	−4.8	0.9	0.8	0.608*a
Clinical + PRS (early fusion)	45	15	15	6	9	67		0.71	62.5	71.4	71.4	62.5	33.9	2.2	4.8	0.019*a
sMRI	45	17	15	6	7	71.1		0.74	70.8	71.4	73.9	68.2	42.1	2.5	6.1	<0.001*a
sMRI + Clinical (stacker)	45	18	11	10	6	63.7		0.71	75	52.4	64.3	64.7	29	1.6	2.5	0.018*a
All modalities (stacker)	45	18	16	5	6	75.6		0.82	75	76.2	78.3	72.7	51	3.1	9.9	0.011*a
Sequential stacker	45	14	17	4	10	69.6		0.74	58.3	81	77.8	63.0	40.7	3.1	0.4	0.014*a
Non-inferiority analysis of LSO-CV vs. P-CV based on 1000 permutations of the site labels																
Clinical	45	10	11	10	14	47.0		0.47	41.7	52.4	50.0	44.0	−6.0	0.9	0.8	0.347*b
Clinical + PRS (early fusion)	45	14	14	7	10	62.5		0.63	58.3	66.7	66.7	58.3	25.0	1.7	2.8	<0.001*b
sMRI	45	17	14	7	7	67.9		0.74	70.8	66.7	69.6	66.7	37.5	2	4.0	0.725*b
PANSS-NS outcome predictor trained on Sham rTMS patients																
Pooled repeated nested cross-validation (P-CV)							BAC_Active_									
Clinical	45	19	4	20	2	53.6	36.0	0.46	90.5	16.7	48.7	66.7	15.4	1.1	1.2	0.257*a
Clinical + PRS (early fusion)	45	13	7	17	8	45.5	47.0	0.52	61.9	29.2	43.3	46.7	−10.0	0.9	0.8	0.770*a
sMRI	45	11	11	13	10	49.1	36.0	0.47	52.4	45.8	45.8	52.4	−1.8	1.0	0.9	0.485*a
sMRI + Clinical (stacker)	45	20	0	24	1	47.6	50.0	0.49	95.2	0	100	1	−54.5	1.0	0.9	0.640*a
All modalities (stacker)	45	21	0	24	0	50	50.0	0.51	100	0	100	1	NaN	1.0	1.0	0.474*a
Leave-one-site-out reported nested cross-validation (LOSO-CV)																
Clinical	45	12	3	21	9	34.8		0.34	57.1	12.5	36.4	25	−38.6	0.7	0.4	0.987*a
Clinical + PRS (early fusion)	45	18	0	24	3	42.9		0.47	85.7	0	42.9	0	−57.1	0.9	0.7	0.864*a
sMRI	45	2	16	8	19	38.1		0.35	9.5	66.7	20	45.7	−34.3	0.3	0.1	0.935*a
sMRI + Clinical (stacker)	45	0	24	0	21	50		0.42	0	100	NaN	53.3	NaN	0	NaN	0.539*a
All modalities (stacker)	45	5	14	10	16	41.1		0.4	23.8	58.3	33.3	46.7	−20	0.6	0.3	0.959*a

Positive/Negative predictions refer to treatment non-response/response after 3 weeks of active or sham rTMS. Hence sensitivity measures the classifiers’ capacity to correctly identify patients with nonresponse to the respective treatment as such. For both the active and sham rTMS predictors 2 types of validation analyses were carried out: Pooled Repeated Nested Cross-Validation (P-CV), and Leave-One-Site-Out Repeated Nested Validation (LOSO-CV), which iteratively trained the respective predictor on 2 out of the 3 sites and then apply the trained predictor to the held-out site. For the active rTMS models further analyses were conducted: (1) analysis of non-inferiority between LOSO-CV and P-CV comparing the effects of reduced training sample size vs. site/scanner effects on classification performance, (2) sham rTMS validation analysis: the active rTMS predictors trained in P-CV was applied to the sham-treated patients to assess whether the predictive models are specific to patients receiving active treatment.

*TP* true positives, *TN* true negatives, *FP* false positives, *FN* false negatives, *BAC* balanced accuracy, *BAC*_*Sham*_ balanced accuracy when predicting Sham treatment group responses using models trained on Active treatment group, *BAC*_*Active*_ balanced accuracy when predicting Active treatment group responses using models trained on Sham treatment group, *AUROC* area under the receiver operating characteristic curve, *Sens* sensitivity, *Spec* specificity, *PPV* positive predictive value, *NPV* negative predictive value, *PSI* Prognostic Summary Index (PSI = PPV + NPV − 100), *LR+* positive likelihood ratio, *DOR* diagnostic odds ratio, *pa* *p* values obtained from permutation analysis of P-CV/LSO-CV predictors trained on respective original label distributions vs. 1000 respective predictors trained on random label permutations, *pb* *p* values obtained from permutation analysis of LSO-CV predictors trained on respective original site label distributions vs. 200 respective predictors trained on random permutations of the patients’ site labels, p* significant *p* value < 0.05. All *p* values are FDR corrected using Benjamini/Hochberg method. *NaN* data not available in the given dataset.

### Neuroanatomical predictive patterns from sMRI model

The neuroanatomical pattern predicting response to the active rTMS treatment involved relatively higher GMD in four areas: (1) cerebellum, (2) dorsomedial and ventromedial prefrontal, frontopolar and cingulate cortices, (3) the insular, opercular, temporopolar and medial temporal cortices and (4) superior and inferior occipital lobe. Higher baseline GMD predicting non-responses was found in the left-hemispheric somatosensory and parietal cortices with extensions to the lateral temporal and premotor structures, as well as in the thalamic nuclei, bilaterally (Fig. 2D). Despite these neuroanatomical predictive patterns having some differences from our previous work, no statistically significant differences were noted between the two patterns (*p*^positive region^ = 0.18, *p*^negative region^ = 0.91) (Supplementary 4.4). Furthermore, we grouped the neuroanatomical predictive patterns according to Yeo atlas brain networks. Default, limbic and frontoparietal networks were particularly related to the prediction of treatment response (Supplementary S22).

### Stacked classifiers performance

Two models were trained and validated using the principle of stacked generalisation. Both stackers achieved higher prediction performance than unimodal classifiers. The sMRI + clinical stacker achieved a BAC of 89.0% (sensitivity: 87.5%, specificity: 90.5%) with a significant BAC increase of 8.9% comparing to the sMRI model (*p* = 0.009). The stacker combining all data modalities achieved a BAC of 89.3% (sensitivity: 83.3%, specificity: 95.2%). It improved BAC by 9.2% when compared to the sMRI model (*p* = 0.009) (Supplementary S11). It also improved prognostic summary index (PSI) from 59.9 to 78.6, positive likelihood ratio from 4.2 to 17.5 and decreased number needed to predict from 1.7 to 1.3 (Table 2).

### Sequential classifier performances

**Fig. 3:**
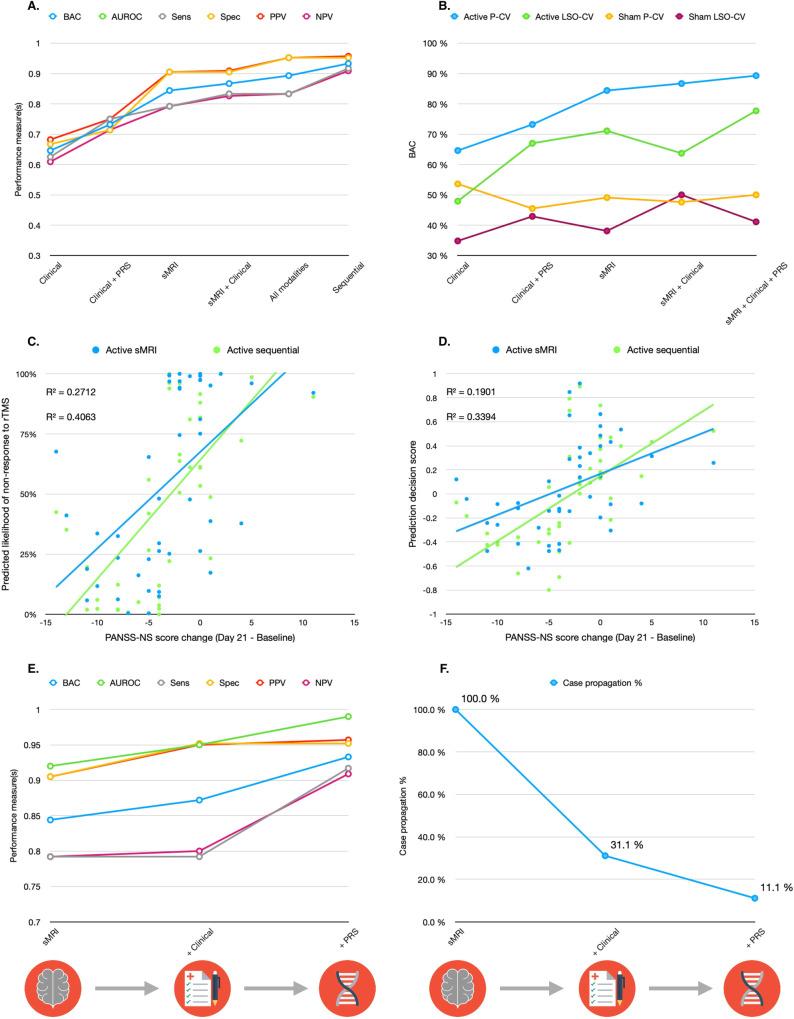
Post-hoc model performance analyses for all RESIS active models. **A** Model performance measures for all RESIS active models. **B** Step-wise BAC performance increase observed in the models in the active treatment group vs. models trained in the sham treatment group in both pooled CV and leave-one-site-out CV (Sequential model not included). **C** Comparison of linear correlations between patients’ predicted likelihood of non-response to rTMS treatment from sMRI model and sequential model and PANSS-NS score reduction from baseline to 21 days after rTMS treatment (upper *R*-squared: sMRI model, lower *R*-squared: sequential model). **D** Comparison of linear correlations between patients’ prediction decision scores from sMRI model and sequential model and PANSS-NS score reduction from baseline to 21 days after rTMS treatment (upper *R*-squared: sMRI model, lower *R*-squared: sequential model). **E** Model performance measures for each prognostic node of the sequential prognostic system (Supplementary S8). **F** The percentage of cases which are propagated at each step of the step-wise sequential model trained on the active treatment group.

Among all RESIS active group models, the optimal sequential model achieved the highest BAC of 93.5% (sensitivity: 91.7%, specificity: 95.2%). The sequential model showed a 50% increase in *R*^2^ value compared to the sMRI model, indicating stronger correlation with PANSS-NS score reduction (sMRI: *R*^2^ = 0.271, Sequential model: *R*^2^ = 0.406, *p* = 0.0002) (Fig. 3C, D) (Supplementary S37). Compared to the sMRI model, the optimal sequence model demonstrated a significant increase in BAC (13.4%), sensitivity (12.5%), and specificity (4.7%) (*p* = 0.0001). Starting with sMRI (BAC: 80.1%, PSI: 59.9), 31.1% of patients progressed to the second model (sMRI + clinical stacker: BAC: 89%, PSI: 77.7), while only 11.1% visited the third model (all-modalities stacker: BAC: 93.5%, PSI: 86.6) (Fig. 3F) (Supplementary S8). Sequential model 6 achieved a comparable BAC of 91.1% (*p* = 0.21) with the optimal sequential model, utilising only the sMRI and clinical models in the sequence, with 46.7% of patients propagated to the second stage clinical model. Sequential model 5 achieved a BAC of 80.7% with two nodes, statistically similar to the sMRI model (*p* = 0.44). The prognostic sequence started with the clinical + PRS model, with 57.8% of patients progressing to the sMRI model (Supplementary S5–10).

### Permutation significance and cross-over validation results

We conducted label permutation tests on all P-CV active group classifiers and showed that their BACs were significant after correcting for multiple comparisons using the false-discovery rate (*p* range: 0.001–0.031). We also conducted feature permutation tests on these models and found that all of the prediction patterns were significant (*p* range: 0.001–0.005), except for the sMRI + Clinical stacker (*p* = 0.89) (Supplementary S13). We applied the active group models to sham-treated patients, the BACs were around chance level for all models (BAC range: 50.0%–53.6%). All models trained on sham-treated patients had BAC values around chance level (BAC range: 45.5%–53.6%) and none was statistically significant (*p* range: 0.257–0.770). When we applied the sham models to active-treated patients, the BACs were also around or below chance level (BAC range: 36%–50%) (Table 2).

### Leave-one-site-out model performance

When evaluating our model’s cross-site generalisability using LOSO-CV, we observed the following BAC performances: (1) Clinical: 47.9%, (2) Clinical + PRS: 67%, (3) sMRI: 71.1%, (4) sMRI + Clinical: 63.7%, (5) all modalities: 77.7%, (6) sequential: 69.6%. Except for the clinical model BAC (*p* = 0.608), all other LSO models’ BACs were significant (*p* range: 0.001–0.019). These performances did not differ from the BACs obtained in the 1000 random permutations of the patients’ site membership in sMRI (*p* = 0.725) and clinical (*p* = 0.347) models, indicating that no residual site effects were present. There was a significant difference between the observed and LSO permuted variant of the clinical + PRS model (*p* < 0.001) due to the fact that 15 patients had no PRS data and the missing PRS were imputed in each training fold using early fusion (Table 2).

### Cross-modalities correlation analyses results

When we correlated the GMD data with clinical features, we found that the superior, middle, inferior and medial frontal gyri showed that the most significant correlations (ROI correlation count = 23), including MADRS items, substance addiction and abuse, as well as PANSS-PS scores, followed by cerebellum (ROI correlation count = 18) and temporal lobe (ROI correlation count = 17) (Supplementary S26). Cerebellar volumes were correlated with MADRS items, GAF score, PANSS-GS and -PS scores as well as substance and alcohol abuse. Temporal lobe volumes were correlated with MADRS items, CGI-S1, PANSS-NS and substance abuse (Supplementary S2,5). Among brain networks, the default network volumes showed the largest number of correlations with clinical features including reduced appetite (MADRS-5), substance addiction and abuse. The limbic network volumes were correlated with apparent sadness (MADRS-1) and reported sadness (MADRS-2). No significant correlations were found between sociodemographic features and GMD (Supplementary S20). The frontal lobe (ROI correlation count = 19) and cerebellum (ROI correlation count = 17) showed the highest number of significant correlations with PRS features. All ROIs within the frontal lobe were correlated with PRS-SZ, except for the medial orbital gyrus which was correlated with PRS-EA. The crus of the cerebellum was correlated with PRS-EA while the vermis was correlated with PRS-SZ (Supplementary S29). In terms of brain networks, frontoparietal and somato-motor networks were correlated with PRS-SZ. No significant correlations were found between brain networks and PRS-EA (Supplementary S32).

### Treatment stratification effects

Supplementary analyses indicated that patients stratified to the response group based on the predictions of our active rTMS models showed significantly higher treatment response rates (sMRI model: 79.2% responders, sMRI + Clinical model: 82.6%, all modalities stacker: 83.3%, sequential model: 90.9%) compared to the original non-stratified patient sample (46.7% responders) (Supplementary S33–S35). We found significant linear correlations between the predicted rTMS response likelihood and PANSS-NS score reduction 21 days after the treatment in all of our active models with *R*² ranging from of 0.20 to 0.41, except for the clinical model. We observed large effect sizes (Cohen’s *d* > 0.80) in patients with a predicted rTMS responsive and medium (Cohen’s *d* < 0.50) to small (Cohen’s *d* < 0.20) effect sizes in patients with non-response prediction in all active-group models. We found no significant correlation between the predicted rTMS response likelihood and PANSS-NS score reduction 21 days after the treatment in the sham models (Supplementary S37).

## Discussion

To our knowledge, this is the first study reporting the successful application of clinical, sociodemographic and PRS-based as well as multimodal machine learning models to the prediction of individual response to rTMS treatment in patients with schizophrenia. We significantly extended the scope of our previous work [6] by incorporating new data domains and multimodal sequential modelling strategies. With the sequential model, we were able to increase the prediction performance of unimodal classifiers from 80.1% to 93.5% and the prognostic certainty increase from +69.6% to +86.5%, compared to our previous work. We observed that individual rTMS treatment responses could be predicted with a BAC of 75.9% using clinical and PRS data. Our methods facilitated robust generalisability to new study sites despite the lower training sample sizes in the LOSO-CV.

The high prediction accuracies achieved in our active group models showed that, despite high inter-individual variability in rTMS treatment responses, there are underlining neuroanatomical, clinical, and genetic patterns which can forecast the likelihood of treatment outcome on an individual level. Moreover, the chance-level prediction results on sham-treated patients confirmed that our active rTMS response models were not only accurate but also therapeutically specific. Our cross-over model validation results further emphasised the therapeutic specificity of our models. This is important because in the RESIS trial, both active and sham-treated groups showed significant PANSS-NS reductions between baseline and 21 days (*p*^active^ = 5.24E−05, *p*^sham^ = 3.00E−06). Therefore, to differentiate the efficacy of active from sham rTMS, it is necessary to apply the same modelling methodology to both groups. Furthermore, the chance-level prediction performances do not suggest that the sham-treated patients have a different pathobiology comparing to the actively treated patients, but only indicate that no general outcome-predictive pattern could be identified for the sham intervention.

Importantly, our study demonstrated that the challenges of diagnostic cost, feasibility and acceptability arising from multimodal prognostic classifiers could be mitigated by using sequential prediction strategies. Despite showing higher prediction accuracies, models utilising data domains such as brain scans, genetic and blood markers may have prohibitive data acquisition and processing costs which may greatly limit their accessibility in the clinical setting [15], particularly in low and middle-income countries. However, current evidence suggests that these multimodal techniques are needed to resolve the disease and treatment course heterogeneity of affective and psychotic disorders potentially caused by the multifactorial nature of these conditions [19, 20]. To overcome this dilemma, our proposed stepwise sequential approach reduces costs by requesting additional data only when necessary for conclusive predictions. For example, our optimal sequential model, stratifying data acquisition into three steps, achieved the highest prediction accuracy while requiring full data acquisition for only 11% of patients. These sequential models would significantly reduce the data acquisition costs compared to their fully stacked counterparts.

Our multi-modal results linking baseline neuroanatomical, clinical, and genetic variations in schizophrenia and rTMS treatment outcome supports the hypothesis proposed by previous research that brain plasticity is a crucial determinant of the effectiveness of brain stimulation approaches such as rTMS. Hasan et al. found that rTMS effectiveness in patients with schizophrenia may depend on the brain’s capacity for mounting structural plasticity responses in the limbic and default mode network (DMN) [9]. In our sMRI model, we found that the neuroanatomical pattern predicting response to active rTMS was particularly associated with relatively higher GMD in the DMN and limbic networks as well as motor-thalamic regions. These findings may suggest that patients with higher GMD in these regions have an increased likelihood for responding to rTMS treatment. In contrast, patients who have higher GMD in sensorimotor regions may not have this advantage. Additionally, impaired anticorrelated coupling between the dorsolateral prefrontal cortex (DLPFC)-based Central Executive Network (CEN) and the medial prefrontal, frontopolar, and medial parietal regions of the DMN have been found in depression and schizophrenia [32, 33]. Studies showed that high-frequency rTMS may attenuate abnormally elevated within-default network connectivity and restore anticorrelated activation patterns of the DMN and CEN [34, 35]. GMD of these regions have also been identified by our sMRI model to be predictive of rTMS treatment response. These regions’ GMDs were also highly correlated with MADRS items which were highly predictive of treatment response in the clinical + PRS model (Supplementary S25). Our results are consistent with these previous findings where the DMN and limbic networks are the most predictive of rTMS treatment response. The high correlation between DMN and limbic networks and clinical variables suggests that the underlining neuroanatomical predictive patterns are reflected in the clinical predictive pattern underlying our model.

We observed that PRS-EA was more predictive of treatment response than PRS-SZ in our clinical + PRS model. PRS-EA has been associated with brain compensatory potential, cognitive abilities, and white matter integrity. Richards et al. [36] showed positive correlations between PRS-EA and cognition in schizophrenia patients, independent of PRS-SZ, suggesting its relevance to cognitive abilities in the context of the disease. Jansen et al. [37] found positive associations between global fractional anisotropy and PRS-EA, suggesting that higher PRS-EA is associated with better white matter integrity, which may contribute to improved treatment response in schizophrenia patients compared to those with lower PRS-EA.

Our study has limitations. Since RESIS is the only multi-site randomised trial investigating rTMS treatment response in schizophrenia patients, no external validation has been possible to date. Given the high prediction accuracies and small sample sizes of our study, large-scale international validation studies are needed to rule out the possibility of overfitting and assess the generalisability of the proposed models beyond our discovery sample. Due to the ancestral outliers, we did not have an equal number of PRS data compared to sMRI and clinical data. This affected our machine learning modelling strategy and therefore, a standalone PRS model could not be incorporated into the multi-modal prognostic system. Our study shows a high predictive value of polygenic scores for education attainment, which is influenced by both sociological and genetic factors. The inclusion of parental education attainment as a highly predictive variable in our clinical + PRS model emphasises this complexity. Therefore, our conclusion about PRS-EA should not be interpreted as a purely genetic signature, but as a complex phenotype influenced by social and economic factors.

Recent studies showed high accuracies (82.5%–95.8%) in predicting responses to pharmacological and electroconvulsive treatment in patients with schizophrenia using functional MRI and electroencephalography [38–42]. In keeping with these findings, our study suggests that brain compensatory potential and neuroplasticity may be predictive of rTMS treatment response. Future studies should explore whether the prediction of rTMS treatment response could be further enhanced using brain connectivity and white matter integrity measurements. This could help solidify our study’s findings and form a more unified explanation of the individual variability in rTMS treatment response in schizophrenia.

In conclusion, our study found that individual response variability to rTMS can be optimally deciphered by integrating phenotypic, neuroimaging, and genetic data using multimodal machine learning strategies. Furthermore, we demonstrated that a stepwise sequential approach can be an effective mitigation strategy which maximises prediction accuracy while controlling costs and diagnostic burden in future precision psychiatry workflows. This approach could improve acceptability and accessibility of such models in the clinical setting. Our study further suggests that rTMS responders may have more adaptive default-mode and limbic networks, thus increasing their response likelihood to rTMS. Multi-site prospective rTMS validation studies and stratified clinical trials covering a larger and more diverse population of patients with schizophrenia recruited in different parts of the world are the mandatory next step to benchmark these findings and further optimise the proposed tools for translation into real-world clinical care.

### Supplementary information

Supplementary Material

## Data Availability

The RESIS dataset used in this paper can be requested from the corresponding co-author with additional approval.

## Code availability

The code for these analyses is available upon request and the active-group models can be accessed for external validation through our online model library (www.proniapredictors.eu).
