# Supplementary material for: Multimodal workflows optimally predict response to repetitive transcranial magnetic stimulation in patients with schizophrenia: a multisite machine learning analysis
Source: Transl Psychiatry. 2024 Apr 25;14:196. doi: 10.1038/s41398-024-02903-1 (PMC11045783; doi:10.1038/s41398-024-02903-1)
Supplement: Supplementary file 1 — Supplementary Material [file 41398_2024_2903_MOESM1_ESM.docx]

**Using Multimodal Data to Predict Response to Repetitive Transcranial Magnetic Stimulation in Patients With Schizophrenia: A Multisite Machine Learning Analysis**

**Supplementary Material**

***Chapters***

*C1. RESIS clinical and sociodemographic data acquisition and pre-processing.............................................. Page 3*

*C2. RESIS genetic data acquisition and PRS calculation................................................................................. Page 4*

*C3. Details of machine learning pipelines...................................................................................................... Page 5*

*C4. Predictive pattern extraction methods using ensemble learning........................................................... Page 13*

*C5. RESIS active Post-hoc cross-modalities correlation analyses................................................................. Page 21*

*C6. RESIS active and sham models post-hoc predicted treatment effects analyses.................................... Page 35*

*References................................................................................................................................................... Page 42*

**Tables & Figures**

*S1. Sociodemographic and clinical data feature definition............................................................................ Page 3*

*S2. Clinical + PRS model early fusion real PRS data vs imputed PRS data T-test results................................ Page 7*

*S3. PRS-only model prediction performances................................................................................................ Page 7*

*S4. All prognostic sequence combinations optimised by the sequential model............................................. Page 9*

*S5. All sequential models’ prediction performances.................................................................................... Page 10*

*S6. All sequential models’ pairwise Z-test results (FDR corrected p-values)................................................ Page 10*

*S7. All sequential models’ pairwise Z-test results (Z.score)......................................................................... Page 10*

*S8. Optimal sequential classifier’s performances on the RESIS Active sample............................................ Page 11*

*S9. Second best sequential classifier’s performances on the RESIS Active sample...................................... Page 11*

*S10. Third best sequential classifier’s performances on the RESIS Active sample....................................... Page 11*

*S11. RESIS active models pairwise Z-test results (FDR corrected p-values)................................................. Page 11*

*S12. RESIS active models pairwise Z-test results (Z-score).......................................................................... Page 12*

*S13. RESIS active and sham models feature and label permutations results.............................................. Page 12*

*S14. ADASYN boosted models prediction performances............................................................................. Page 13*

*S15. RESIS active treated group clinical + PRS model predictive pattern.................................................... Page 15*

*S16. Gray Matter Density predictive pattern extracted from sMRI model of our previous work................ Page 16*

*S17. Gray Matter Density predictive pattern extracted from sMRI model of the current study.................. Page 17*

*S18. Significant ROI for predicting responder to rTMS treatment of our previous work............................. Page 17*

*S19. Significant ROI for predicting responder to rTMS treatment of the current study............................... Page 18*

*S20. Significant ROI for predicting non-responder to rTMS treatment of our previous work...................... Page 19*

*S21. Significant ROI for predicting non-responder to rTMS treatment of the current study........................ Page 20*

*S22. Significant brain networks for predicting responder to rTMS treatment of the current stud.............. Page 21*

*S23. Significant brain networks for predicting non-responder to rTMS treatment of the current study..... Page 21*

*S24. Scatter plot prediction comparisons between sMRI and clinical+PRS models..................................... Page 22*

*S25. Significant correlations between clinical features and ROIs................................................................ Page 22*

*S26. Significant correlations between clinical features and ROIs, summed by brain regions...................... Page 27*

*S27. Significant correlations between clinical features and ROIs, summed by clinical features.................. Page 27*

*S28. Significant correlations between clinical features and brain networks................................................ Page 28*

*S29. Significant correlations between PRS features and ROIs..................................................................... Page 29*

*S30. Significant correlations between PRS features and ROIs, summed by brain regions........................... Page 33*

*S31. Significant correlations between PRS features and ROIs, summed by PRS features............................ Page 33*

*S32. Significant correlations between PRS features and brain networks.................................................... Page 34*

*S33. Line plot of the response rates of all active models………………………………………………………………………… Page 36*

*S34. response counts and rates of all active models…………………….………………………………………………………… Page 37*

*S35. Chi2 comparison of treatment responses from all active models…………………….……………………………… Page 37*

*S36. Scatter plot linear correlations between predicted likelihood and PANSS-NS reduction..................... Page 38*

*S37. Linear correlations and effect sizes between predicted likelihood and PANSS-NS reduction.............. Page 39*

*S38. Scatter plot linear correlations predicted likelihood and PANSS-NS reduction (longitudinal)............. Page 40*

*S39. Linear correlations and effect sizes predicted likelihood and PANSS-NS reduction (longitudinal)……. Page 41*

#### **C1. RESIS Clinical and sociodemographic data acquisition and pre-processing**

To create a predictive model, only baseline data were used in the machine learning analyses. We condensed the 106 clinical, comorbidity and sociodemographic variables into 25 features in order to increase the robustness of the classifiers. We summed the education years of both parents into one feature called ‘education parents’. We summed PANSS P, N, G questionnaire items into 3 features containing the total scores of each. We summed CDSS questions into one single score.

The final clinical and sociodemographic features include the following: 16 clinical features: PANSS-PS (positive scores), PANSS-NS (negative scores), PANSS-GS (general scores), Calgary Depression Scale for Schizophrenia score (CDSS), Clinical Global Impressions: Sickness Severity score (CGI1), Global Assessment of Functioning score (GAF), Montgomery-Åsberg Depression Rating Scale items (MADRS 1 to 10); 4 comorbidity features: Alcohol abuse, Alcohol addiction, Substance abuse, Substance addiction. The co-morbidities are based on the previous life-time history of alcohol and substance abuse and addiction prior to the study. All participants of the RESIS study have no on-going alcohol and substance abuse and addiction during the study recruitment and duration of the treatment, as they are part of the study exclusion criteria (Wobrock et al. 2015). 5 socio-demographic features: Marital status (married / not married), Employment status (working / not working), Housing status (live alone / live with someone), Years of education, Sum of years of education from both parents.

***Table S1.*** *Sociodemographic and clinical data feature definition.*

***Abbreviations:*** *PANSS: Positive and Negative Syndrome Scale, CDSS: Calgary Depression Scale for Schizophrenia score, CGI-S1: Clinical Global Impressions: Sickness Severity score, GAF: Global Assessment of Functioning score, MADRS:* *Montgomery–Åsberg Depression Rating Scale.*

| **Feature** | **Definition** |
| --- | --- |
| **Marital status** | Marital / relationship status |
| **Employment status** | Employment status |
| **Housing status** | Housing status |
| **Education (y)** | Years of education received by the subject |
| **Education parents** | Sum of the years of education received by both parents of the subject |
| **Alcohol abuse** | Comorbidity: Alcohol abuse |
| **Alcohol addiction** | Comorbidity: Alcohol addiction |
| **Substance abuse** | Comorbidity: Substance abuse |
| **Substance addiction** | Comorbidity: Substance addiction |
| **PANSS-PS** | Sum of PANSS Positive items scores at Baseline |
| **PANSS-NS** | Sum of PANSS Negative items scores at Baseline |
| **PANSS-GS** | Sum of PANSS General items scores at Baseline |
| **CDSS** | Sum of Calgary Depression Scale for Schizophrenia items scores at Baseline |
| **CGI-S1** | Clinical Global Impressions (CGI-S 1): Sickness Severity at Baseline |
| **GAF** | Global Assessment of Functioning at Baseline |
| **MADRS-1** | MADRS-1: Apparent Sadness at Baseline |
| **MADRS-2** | MADRS-2: Reported Sadness at Baseline |
| **MADRS-3** | MADRS-3: Inner Tension at Baseline |
| **MADRS-4** | MADRS-4: Reduced Sleep at Baseline |
| **MADRS-5** | MADRS-5: Reduced Appetite at Baseline |
| **MADRS-6** | MADRS-6: Concentration Difficulties at Baseline |
| **MADRS-7** | MADRS-7: Lassitude at Baseline |
| **MADRS-8** | MADRS-8: Inability to feel at Baseline |
| **MADRS-9** | MADRS-9: Pessimistic Thoughts at Baseline |
| **MADRS-10** | MADRS-10: Suicidal Thoughts feel at Baseline |

#### **C2. RESIS genetic data acquisition and PRS calculation**

Genetic information was obtained from all patients, including both Active and Sham treatment groups. DNA was extracted from peripheral blood samples using automated DNA extraction with the chemagic Magnetic Separation Module I (Chemagen Biopolymer-Technologie, Baesweiler, Germany). All samples were genotyped on the Infinium PsychArray-24 BeadChip (Illumina, San Diego, CA, USA).

SNP-level quality control (QC) included the removal of variants with a call rate <98%, significant deviations from Hardy–Weinberg equilibrium (HWE) (p < 0.001), or minor allele frequency (MAF) < 0.1%. In the individual-level QC, data were removed to eliminate duplicates, sex mismatches, cryptic relatives (PI-HAT > 0.125), samples with an individual genotyping rate >98%, or heterozygosity rate exceeding 3 SD from the mean. Population stratification was assessed using multidimensional scaling (MDS) components analysis on the pairwise genomic relationship matrix. Individuals with genotypes not clustered with 1000 Genomes Project EUR super-populations were removed from the analyses. Taken together, 15 active treated patients and 15 sham treated patients were not included in genetic analyses due to genetic ancestry outliers or because they did not survive QC. These patients entered the machine learning analyses without corresponding PRS scores. 30 active treated patients and 30 sham treated patients retained their corresponding PRS scores for the subsequent analyses.

After QC, pre-phasing was performed using SHAPEIT [^18^](https://www.zotero.org/google-docs/?ocoQsX) and imputation was conducted using IMPUTE2 [^19^](https://www.zotero.org/google-docs/?a7NRY9). The phase 3 integrated variant dataset from the 1000 Genomes Project [^20^](https://www.zotero.org/google-docs/?0ikbUA) was used as the reference panel. After imputation, variants with a low information score (INFO < 0.9) or frequency (MAF < 1%) were removed from the analyses. PRSs were computed using a recently developed Bayesian regression and continuous shrinkage priors method (PRS-CS) [^21^](https://www.zotero.org/google-docs/?PwXPW5). The posterior effect sizes of SNPs were inferred using information from schizophrenia [^22^](https://www.zotero.org/google-docs/?eevcfO) and educational attainment [^23^](https://www.zotero.org/google-docs/?yAE1v6) GWAS summary statistics and an external linkage disequilibrium reference (1000 Genomes Project phase 3, EUR super-populations), through principles of joint multivariate modelling. This method generates different scores based on different assumptions of polygenicity and also estimates the polygenicity of the trait with an auto *φ* parameter. All of the PRS scores were normalised using z-score transformation. 12 PRS scores are used in the machine learning analyses: 6 Education Attainment PRS (*φ* values 1 to 1e-6) and 6 Schizophrenia PRS (*φ* values 1 to 1e-6).

#### **C3. Details of machine learning pipelines**

Following our previous work and recommended for predictive modelling, we employed repeated nested cross-validation with 10 permutations X 20 folds at the outer CV cycles, and a 1 X 19 folds at the inner CV cycle to achieve unbiased estimation of model generalisability to new patients. All models are trained using the linear kernel Support Vector Machine (SVM) algorithm. This algorithm finds the optimal decision boundary that best predicts the rTMS treatment outcome of the patients. The linear kernel SVM is robust for generating models in a high dimension low sample size (big-P, little-N) situation and is less prone to overfitting. All SVM models generated from an inner CV cycle were combined into an ensemble classifier, where the final prediction score is calculated based on majority voting of all sub classifiers. The ensemble classifier was then applied to the respective outer CV data to evaluate the performance. This process was repeated across all outer CV folds of the repeated nested CV design. We employed four different preprocessing pipelines to cater for the different characteristics of the 6 different models. The pipelines are embedded in each inner cycle of the CV structure to avoid information leakage.

**3.1 sMRI data preprocessing**

The preprocessing for the sMRI model follows our previous work (Koutsouleris et al., 2018). After the preprocessing steps described in “sMRI imaging data acquisition and pre-processing”, the GMD matrix were standardised based on their voxel.level mean and SDs. Then the data were site-adjusted using variance extraction multivariate correction method [^24^](https://www.zotero.org/google-docs/?bqUXK1). In this method, firstly Principal Component Analysis (PCA) reduced the GMD matrix to 20-25 principal components (PCs) while retaining 80% of variance from the GMD. Then, analysis of variance (ANOVA) fitted 3 predictors for each of the 3 site memberships in the training dataset. PCs explaining site membership design matrices with R2 > 0.16 were removed. This step removes the site-effects. Finally, processed data were scaled feature-wise between 0 and 1. This is the final preprocessed data that entered the SVM classifiers in each inner CV fold. The same preprocessing pipeline is also applied to the respective test and validation data in order to evaluate model performances.

**3.2 clinical and PRS data preprocessing**

The preprocessing of clinical model and clinical + PRS model follow a different pipeline. Firstly, the features were scaled feature-wise between 0 and 1, and non-informative features were pruned. Then all missing data were imputed using the median of 7 nearest neighbours and interpolated using euclidean distance. Next, age, sex and site effects are corrected using partial correlation linear regression method. Finally, the data were scaled feature-wise once more before model training. It is important to note that the clinical + PRS model combines the two data domains using early fusion, which is a simple concatenation of the features from the two modalities into 1 input matrix before entering preprocessing.

**3.3 Early fusion imputation validity test**

In order to combine clinical and PRS modalities despite the fact that 15 patients in both RESIS active and sham cohorts have no PRS data available, we used imputation within early fusion to generate the missing PRS data using clinical data from the same patient. Since this imputation is cross-modalities, an extra validity test is conducted in order to verify that the imputed PRS data are not significantly different to the real PRS data present. The validation is done by comparing statistical significance between the real PRS data and imputed PRS data using independent T-test for every permutation and fold in both inner and outer CV, resulting in 3800 comparisons (10*20*1*19). The p-values from T-tests are FDR-corrected. There is almost no significant differences found between real PRS data and imputed PRS data in all folds and permutations, indicating the imputation method used to generate missing PRS data is reliable [Supplement S2].

***Table S2.*** *Clinical + PRS model early fusion real PRS data vs imputed PRS data T-test results. The significant count is the number of comparisons between real PRS and imputed PRS where there is a significant difference found (p(fdr)<.05). Significant % shows the percentage of significant differences in all 3800 comparisons. It is evident in this table that the highest significant differences percentage is only 0.5%.*

| **Feature** | **t mean** | **t std** | **p (fdr) mean** | **p (fdr) std** | **total count** | **significant count** | **significant %** |
| --- | --- | --- | --- | --- | --- | --- | --- |
| **PRS SZ (phi:1e-1)** | 1.54 | 0.89 | 0.67 | 0.26 | 3800 | 1 | 0 |
| **PRS SZ (phi:1e-2)** | 0.96 | 0.73 | 0.83 | 0.18 | 3800 | 0 | 0 |
| **PRS SZ (phi:1e-3)** | 0.05 | 0.72 | 0.89 | 0.15 | 3800 | 11 | 0.3 |
| **PRS SZ (phi:1e-4)** | -0.92 | 0.66 | 0.85 | 0.18 | 3800 | 19 | 0.5 |
| **PRS SZ (phi:1e-5)** | -0.75 | 0.43 | 0.88 | 0.14 | 3800 | 0 | 0 |
| **PRS SZ (phi:1e-6)** | -0.15 | 0.37 | 0.92 | 0.1 | 3800 | 0 | 0 |
| **PRS EA (phi:1e-1)** | 0.24 | 0.52 | 0.91 | 0.11 | 3800 | 0 | 0 |
| **PRS EA (phi:1e-2)** | 0.04 | 0.44 | 0.91 | 0.11 | 3800 | 0 | 0 |
| **PRS EA (phi:1e-3)** | 0.03 | 0.41 | 0.91 | 0.1 | 3800 | 0 | 0 |
| **PRS EA (phi:1e-4)** | 0.23 | 0.38 | 0.91 | 0.1 | 3800 | 0 | 0 |
| **PRS EA (phi:1e-5)** | 0.1 | 0.38 | 0.91 | 0.1 | 3800 | 0 | 0 |
| **PRS EA (phi:1e-6)** | 0.44 | 0.45 | 0.9 | 0.11 | 3800 | 0 | 0 |

**3.4 PRS-only classifier**

A separate PRS-only classifier has been trained differentiate response vs non-response in RESIS active patients. However, because only 30 participants out of 45 have PRS data, the classifier cannot be integrated into hierarchical stacker modelling process and the performance of the classifier cannot be fairly compared with the other classifiers trained using all 45 participants. The performance of the PRS-only classifier is listed below [Supplement S3].

***Table S3****. PRS-only model prediction performances with random pooled CV scheme. The PRS only model is not included in the main article because only 30 out of 45 participants from RESIS active group have PRS data, therefore the PRS model cannot be incorporated into the hierarchical stacker models as well as sequential model. The prediction performances of the PRS model is also not comparable to the other models.*

***Abbreviations:*** *TP: true positives, TN: true negatives,  FP: false positives, FN: false negatives, BAC, balanced accuracy; AUROC: Area under the Receiver Operating Characteristic curve, Sens: Sensitivity, Spec: Specificity, PPV: Positive Predictive Value, NPV: Negative Predictive Value, PSI: Prognostic Summary Index (PSI = PPV + NPV − 100), LR+: Positive Likelihood ratio, DOR: Diagnostic Odds Ratio.*

|  | **N** | **TP** | **TN** | **FP** | **FN** | **BAC** | **AUROC** | **Sens** | **Spec** | **PPV** | **NPV** | **PSI** | **LR+** | **DOR** |
| --- | --- | --- | --- | --- | --- | --- | --- | --- | --- | --- | --- | --- | --- | --- |
| **PRS (P-CV)** | 30 | 10 | 9 | 4 | 7 | 64 | 0.64 | 58.8 | 69.2 | 71.4 | 56.2 | 27.7 | 1.9 | 3.7 |

**3.5 Stacked and sequential models preprocessing**

For the two stacker models as well as the sequential model, the only preprocessing step is to scale the base learner decision scores feature-wise between 0 and 1. The sequential model’s preprocessing steps include scaling base learner decision scores feature-wise between -1 and 1, and then imputed the missing values using the median of 7 nearest neighbours and interpolated using euclidean distance.

**3.6 Case propagation-based sequential prognostic algorithm**

Following our previous work (Koutsouleris et al., 2021), we employed a sequential stacking algorithm in NeuroMiner to generate cost-effective prognostic machine learning tools for clinical care. The algorithm optimises the application sequence of predictive models that maximises prognostic accuracy and reduces the per-case examinations needed to achieve this performance. More specifically, the objective of the sequence optimizer is to search through a space of every combination of stacking possibilities, while intelligently choosing individuals for whom prognostic (or diagnostic) accuracy would improve from the addition of new modalities in the stack. In our study, the combination of stacking possibilities was divided into three different sequential nodes that represent the prediction scores based on the three modalities (sMRI, clinical, PRS) as well as their respective stackers. A total of 7 candidate prognostic combinations are available from our sequential nodes compositions [Supplement S4].

To identify individuals who need additional sequences, we employed a method that maximizes the decision score margin between the cases of the opposite classes within specific percentile windows. Specifically, for a given predictive sequence, the algorithm first ranks the training cases according to their decision scores in the sequential node, and then, starting from the decision boundary with a 5%-step width, determines the optimal upper and lower decision score percentiles for which case propagation to the next prediction node would maximize the decision score margin between cases of opposite classes. Establishing increasing percentile windows around the decision boundary is expected to place a gradient from individuals with most ambiguous decision scores to individuals with a very unequivocally predicted class membership. This procedure is repeated across all subsequent prognostic nodes of the given sequence, for all prognostic sequences to be tested, and for each CV1 data partition so that a performance profile can be computed across the three-dimensional hyper-parameter cube of the algorithm, i.e. the sequence pool, the lower and the upper case propagation percentiles. In the current study, we defined 7 candidate multimodal sequences and the lower (- from anchor) and upper (+ from anchor) thresholds around the decision boundary ([±10%, ±20%, ±30%, ±40%, ±50%] of cases, resulting in a total of 7 * 5 * 5 = 175 hyper-parameter combinations. NeuroMiner finds the optimal sequence and hyper-parameter combination across all CV folds and permutations and returns the optimal prognostic sequence to be implemented into clinical application. Additionally, we listed the performance of all sequence combination models in Supplement S5. In which, three sequential models achieved higher BAC than the best unimodal classifier (sMRI model, BAC=80.1%). The performance of each prognostic node in the 3 best performing sequential models can be found in Supplement S8, S9 and S10.

When applying the sequential model, an individual test case will start with the first model node and will only propagate to the second model node if the prediction result from the first model lies outside of the optimised lower and upper propagation thresholds. This method will greatly reduce the data domains needed for prediction meanwhile maintaining a similar prediction accuracy in large scale application (Koutsouleris et al., 2021).

***Table S4.*** *All prognostic sequence combinations optimised by the sequential model, the winning sequence is marked as red.*

| **Sequential model** | **Node 1** | **Node 2** | **Node 3** |
| --- | --- | --- | --- |
| **1** | Clinical (unimodal) -> | Clinical + PRS (early fusion unimodal) | *End of sequence* |
| **2** | Clinical (unimodal) -> | Clinical + PRS (early fusion unimodal ) -> | sMRI (all modalities stacker) |
| **3** | Clinical (unimodal) -> | sMRI + Clinical (stacker) | *End of sequence* |
| **4** | Clinical (unimodal) -> | sMRI + Clinical (stacker) -> | PRS (all modalities stacker) |
| **5** | Clinical + PRS -> | sMRI (all modalities stacker) | *End of sequence* |
| **6** | sMRI (unimodal) -> | sMRI + Clinical (stacker) | *End of sequence* |
| **7** | **sMRI (unimodal) ->** | **sMRI + Clinical (stacker) ->** | **PRS (all modalities stacker)** |

***Table S5.*** *All prognostic sequential models’ prediction performances, sequential models which achieved higher or comparable BAC than the sMRI unimodal classifier (the best performing unimodal classifier) is coloured green. The optimal sequence is coloured in red.*

***Abbreviations:*** *refer to supplement S3.# needed to predict: number needed to predict, interpreted as the number of patients who need to be examined in the patient population in order to correctly predict the diagnosis of one person.*

| **Sequential models** | **N** | **TP** | **TN** | **FP** | **FN** | **BAC** | **AUROC** | **Sens** | **Spec** | **PPV** | **NPV** | **PSI** | **LR+** | **# needed to predict** | **DOR** |
| --- | --- | --- | --- | --- | --- | --- | --- | --- | --- | --- | --- | --- | --- | --- | --- |
| 1 | 45 | 17 | 14 | 7 | 7 | 68.8 | 0.74 | 70.8 | 66.7 | 70.8 | 66.7 | 37.5 | 2.1 | 2.7 | 4.5 |
| 2 | 45 | 18 | 14 | 7 | 6 | 70.8 | 0.73 | 75 | 66.7 | 72 | 70 | 42 | 2.2 | 2.4 | 5.1 |
| 3 | 45 | 18 | 15 | 6 | 6 | 73.2 | 0.75 | 75 | 71.4 | 75 | 71.4 | 46.4 | 2.6 | 2.2 | 6.9 |
| 4 | 45 | 18 | 15 | 6 | 6 | 73.2 | 0.75 | 75 | 71.4 | 75 | 71.4 | 46.4 | 2.6 | 2.2 | 6.9 |
| **5** | **45** | **17** | **19** | **2** | **7** | **80.7** | **0.83** | **70.8** | **90.5** | **89.5** | **73.1** | **62.6** | **7.4** | **1.6** | **55.3** |
| **6** | **45** | **22** | **19** | **2** | **2** | **91.1** | **0.95** | **91.7** | **90.5** | **91.7** | **90.5** | **82.1** | **9.6** | **1.2** | **92.6** |
| **7** | **45** | **22** | **20** | **1** | **2** | **93.5** | **0.95** | **91.7** | **95.2** | **95.7** | **90.9** | **86.6** | **19.2** | **1.2** | **370.6** |

**3.7 Performance comparison between all sequential models using RESIS active-treated samples**

We used Z-test on all 9 sequential models trained on RESIS active treated samples to compare if the BAC performances of the models are significantly different. All p-values are FDR corrected [Supplement S6].

***Table S6.*** *All sequential models pairwise Z-test results (FDR corrected p-values).*

***Bold values*** *indicate statistical significance (p<0.05).*

| **Sequential models** | **Sequence 1** | **Sequence 2** | **Sequence 3** | **Sequence 4** | **Sequence 5** | **Sequence 6** | **Sequence 7** |
| --- | --- | --- | --- | --- | --- | --- | --- |
| **Sequence 1** | .. | 0.34 | *0.2* | *0.2* | ***6.00E-03*** | **8.93E-08** | **1.57E-09** |
| **Sequence 2** | 0.34 | .. | 0.314 | 0.314 | **1.90E-02** | **5.14E-07** | **1.34E-08** |
| **Sequence 3** | 0.2 | 0.314 | .. | 1 | 0.055 | **5.16E-06** | **1.26E-07** |
| **Sequence 4** | 0.2 | 0.314 | 1 | .. | 0.055 | **5.16E-06** | **1.26E-07** |
| **Sequence 5** | **6.00E-03** | **1.90E-02** | 0.055 | 0.055 | .. | **3.00E-03** | **2.00E-04** |
| **Sequence 6** | **8.93E-08** | **5.14E-07** | **5.16E-06** | **5.16E-06** | **3.00E-03** | .. | 0.21 |
| **Sequence 7** | **1.57E-09** | **1.34E-08** | **1.26E-07** | **1.26E-07** | **2.00E-04** | 0.21 | .. |

***Table S7.*** *All sequential models pairwise Z-test results (Z-score).*

| **Sequential models** | **Sequence 1** | **Sequence 2** | **Sequence 3** | **Sequence 4** | **Sequence 5** | **Sequence 6** | **Sequence 7** |
| --- | --- | --- | --- | --- | --- | --- | --- |
| **Sequence 1** | .. | -0.44 | *-0.97* | *-0.97* | *-2.74* | -5.57 | -6.32 |
| **Sequence 2** | -0.44 | .. | -0.53 | -0.53 | -2.31 | -5.17 | -5.93 |
| **Sequence 3** | *-0.97* | -0.53 | .. | 0.00 | -1.78 | -4.67 | -5.45 |
| **Sequence 4** | *-0.97* | -0.53 | 0.00 | .. | -1.78 | -4.67 | -5.45 |
| **Sequence 5** | *-2.74* | -2.31 | -1.78 | -1.78 | .. | -2.99 | -3.82 |
| **Sequence 6** | -5.57 | -5.17 | -4.67 | -4.67 | -2.99 | .. | -0.90 |
| **Sequence 7** | -6.32 | -5.93 | -5.45 | -5.45 | -3.82 | -0.90 | .. |

***Table S8.*** *Optimal sequential classifier’s performances on the RESIS Active sample. The sequential model starts with the sMRI model, followed by the clinical model if the sMRI model is not able to derive a conclusive result, followed by the clinical + PRS model if the clinical model is not able to derive a conclusive result.*

***Abbreviations:*** *refer to supplement S3.P-value:* *P-value obtained from permutation analysis of P-CV predictors trained on respective original label distributions vs 1000 respective predictors trained on random label permutations,* *P*: significant P-value < 0.05, Case prop: percentage of cases which propagated to the next step of the sequential model.*

| **Sequential**  **nodes** | **N** | **TP** | **TN** | **FP** | **FN** | **BAC** | **AUROC** | **Sens** | **Spec** | **PPV** | **NPV** | **PSI** | **LR+** | **DOR** | **P-value** | **Case prop** |
| --- | --- | --- | --- | --- | --- | --- | --- | --- | --- | --- | --- | --- | --- | --- | --- | --- |
| **sMRI** | 45 | 19 | 17 | 4 | 5 | 80.1 | 0.85 | 79.2 | 81 | 82.6 | 77.3 | 59.9 | 4.2 | 17.3 | <0.001* | 100.0% |
| **+ Clinical** | 45 | 21 | 19 | 2 | 3 | 89 | 0.92 | 87.5 | 90.5 | 91.3 | 86.4 | 77.7 | 9.2 | 84.4 | <0.001* | 31.1% |
| **+ PRS** | 45 | 22 | 20 | 1 | 2 | 93.5 | 0.99 | 91.7 | 95.2 | 95.7 | 90.9 | 86.6 | 19.2 | 370.6 | <0.001* | 11.1% |

***Table S9.*** *Second best sequential classifier’s performances on the RESIS Active sample. The sequential model starts with the sMRI model, followed by the clinical model if the sMRI model is not able to derive a conclusive result.*

***Abbreviations:*** *refer to supplement S5.*

| **Sequential nodes** | **N** | **TP** | **TN** | **FP** | **FN** | **BAC** | **AUROC** | **Sens** | **Spec** | **PPV** | **NPV** | **PSI** | **LR+** | **DOR** | **P-value** | **Case prop** |
| --- | --- | --- | --- | --- | --- | --- | --- | --- | --- | --- | --- | --- | --- | --- | --- | --- |
| sMRI | 45 | 19 | 17 | 4 | 5 | 80.1 | 0.85 | 79.2 | 81 | 82.6 | 77.3 | 59.9 | 4.2 | 17.3 | <0.001*a | 100% |
| + Clinical | 45 | 22 | 19 | 2 | 2 | 91.1 | 0.95 | 91.7 | 90.5 | 91.7 | 90.5 | 82.1 | 9.6 | 92.6 | <0.001*a | 46.7% |

***Table S10.*** *Third best sequential classifier’s performances on the RESIS Active sample. The sequential model starts with the Clinical+PRS model, followed by the sMRI model if the Clinical+PRS model is not able to derive a conclusive result.*

***Abbreviations:*** *refer to supplement S5.*

| **Sequential nodes** | **N** | **TP** | **TN** | **FP** | **FN** | **BAC** | **AUROC** | **Sens** | **Spec** | **PPV** | **NPV** | **PSI** | **LR+** | **DOR** | **P-value** | **Case prop** |
| --- | --- | --- | --- | --- | --- | --- | --- | --- | --- | --- | --- | --- | --- | --- | --- | --- |
| Clinical+PRS | 45 | 17 | 17 | 4 | 7 | 75.9 | 0.77 | 70.8 | 81 | 81 | 70.8 | 51.8 | 3.7 | 13.8 | 0.001*a | 100% |
| + sMRI | 45 | 17 | 19 | 2 | 7 | 80.7 | 0.83 | 70.8 | 90.5 | 89.5 | 73.1 | 62.6 | 7.4 | 55.3 | <0.001*a | 57.8% |

**3.8 Performance comparison between all trained models using RESIS active dataset**

We used Z-test on all sub models of each ensemble model to compare if the BAC performances of all models trained on RESIS active patients are significantly different. All p-values are FDR corrected [Supplement S11].

***Table S11.*** *RESIS active models pairwise Z-test results (FDR corrected p-values).*

******* *indicate statistical significance (p<0.05).*

| **Model** | **Clinical** | **Clinical + PRS** | **sMRI** | **sMRI + Clinical** | **All** | **Sequential (optimal)** |
| --- | --- | --- | --- | --- | --- | --- |
| **Clinical** | .. | 0.009* | 0.0005* | 1.86E-08* | 1.69E-08* | 9.52E-12* |
| **Clinical + PRS** | 0.009* | .. | 0.166 | 0.0005* | 0.0005* | 1.90E-06* |
| **sMRI** | 0.0005* | 0.166 | .. | 0.009* | 0.009* | 0.0001* |
| **sMRI + Clinical** | 1.86E-08* | 0.0005* | 0.009* | .. | 0.46 | 0.07 |
| **All** | 1.69E-08* | 0.0005* | 0.009* | 0.46 | .. | 0.08 |
| **Sequential (optimal)** | 9.52E-12* | 1.90E-06* | 0.0001* | 0.07 | 0.08 | .. |

***Table S12.*** *RESIS active models pairwise Z-test results (Z-score).*

| **Model** | **Clinical** | **Clinical + PRS** | **sMRI** | **sMRI + Clinical** | **All** | **Sequential (optimal)** |
| --- | --- | --- | --- | --- | --- | --- |
| **Clinical** | .. | -2.47 | -3.47 | -5.78 | -5.86 | -7.1 |
| **Clinical + PRS** | 2.47 | .. | -1.01 | -3.44 | -3.53 | -4.89 |
| **sMRI** | 3.47 | 1.01 | .. | -2.46 | -2.56 | -3.96 |
| **sMRI + Clinical** | 5.78 | 3.44 | 2.46 | .. | -0.1 | -1.59 |
| **All** | 5.86 | 3.53 | 2.56 | 0.1 | .. | -1.5 |
| **Sequential (optimal)** | 7.1 | 4.89 | 3.96 | 1.59 | 1.5 | .. |

***Table S13.*** *RESIS active and sham models feature and label permutations results.*

***Abbreviations:*** *refer to supplement S3. P (label perm): FDR-corrected P-values obtained from permutation analysis of P-CV predictors trained on respective original label distributions vs 1000 respective predictors trained on random label permutations. P (feature perm): FDR-corrected P-values obtained from permutation analysis of P-CV predictors trained on respective original feature distributions vs 1000 respective predictors trained on random feature value permutations on all features. P*: significant P-value < 0.05. All P values are FDR corrected using Benjamini/Hochberg method.*

| **Active models** | **BAC** | **AUROC** | **P (label perm)** | **P (feature perm)** |
| --- | --- | --- | --- | --- |
| **Clinical** | 64.6 | 0.69 | .031* | .005* |
| **Clinical + PRS** | 73.2 | 0.78 | .001* | <.001* |
| **sMRI** | 84.4 | 0.92 | <.001* | <.001* |
| **sMRI + Clinical** | 86.7 | 0.94 | <.001* | .89 |
| **All stacker** | 89.3 | 0.96 | <.001* | <.001* |
| **Sham models** |  |  |  |  |
| **Clinical** | 53.6 | 0.46 | .257 | .195 |
| **Clinical + PRS** | 45.5 | 0.52 | .770 | .509 |
| **sMRI** | 49.1 | 0.47 | .485 | .447 |
| **sMRI + Clinical** | 47.6 | 0.49 | .640 | .691 |
| **All stacker** | 50 | 0.51 | .474 | .508 |

**3.9 Leave-site-out models using ADASYN to artificially boost sample size to equalise imbalance between sites**

As a way to deal with the site sample imbalance between the three sites in RESIS dataset, we employed ADASYN to artificially boost sample size to equalise the imbalance between sites (He et al., 2008). ADASYN is integrated into NeuroMiner and we trained sMRI, clinical, clinical+PRS, sMRI+clinical and all modalities models with the exact same pipelines and settings as the standard models using RESIS active treated data with the only alteration been the addition of ADASYN technique in the pre-processing pipeline. The model performances can be found in the table below [Supplement S14]. The ADASYN boosted models performed worse than the original models without ADASYN and therefore is not used for further analyses.

***Table S14.*** *ADASYN boosted models prediction performances using RESIS active treated data and leave-site-out repeated CV scheme.*

***Abbreviations:*** *refer to supplement S3.*

| **ML model** | **N** | **TP** | **TN** | **FP** | **FN** | **BAC** | **AUROC** | **Sens** | **Spec** | **PPV** | **NPV** | **PSI** | **LR+** | **DOR** |
| --- | --- | --- | --- | --- | --- | --- | --- | --- | --- | --- | --- | --- | --- | --- |
| **sMRI** | 45 | 16 | 14 | 7 | 8 | 66.7 | 0.73 | 66.7 | 66.7 | 69.6 | 63.6 | 33.2 | 2 | 4 |
| **Clinical** | 45 | 7 | 12 | 9 | 17 | 43.2 | 0.5 | 29.2 | 57.1 | 43.8 | 41.4 | -14.9 | 0.7 | 0.5 |
| **Clinical+PRS** | 45 | 8 | 15 | 6 | 16 | 52.4 | 0.57 | 33.3 | 71.4 | 57.1 | 48.4 | 5.5 | 1.2 | 1.4 |
| **sMRI + Clinical (stacker)** | 45 | 19 | 11 | 10 | 5 | 65.8 | 0.73 | 79.2 | 52.4 | 65.5 | 68.8 | 34.3 | 1.7 | 2.8 |
| **All modalities (stacker)** | 45 | 15 | 15 | 6 | 9 | 67 | 0.75 | 62.5 | 71.4 | 71.4 | 62.5 | 33.9 | 2.2 | 4.8 |

**C4. Predictive pattern extraction methods using ensemble learning**

Apart from the conventional feature weights extraction and feature vs prediction spearman correlation analysis, 2 additional computational approaches, as implemented in NeuroMiner, were used to visualize the predictive patterns elements in the models.

**4.1. Cross-validation ratio (CVR)**

We calculated the pattern element *stability*, termed cross-validation ratio (CVR), by computing the mean and standard error of all SVM weight vectors concatenated across the entire nested cross- validation structure. This cross-validation ratio as a measure for pattern stability was inspired by the bootstrap ratio commonly used in the Partial Least Squares literature and described in (Krishnan et al., 2011). Similarly to the bootstrap ratio, the CVR of pattern element 𝑗, be it a clinical-cognitive variable, a polygenic risk score, an image voxel or the decision score of a unimodal risk calculator, was defined as:


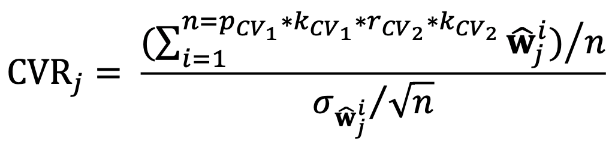


Where 𝑛 is the size of the SVM ensemble, 𝑝_𝐶𝑉1_ is the number of CV1 permutations, 𝑘_𝐶𝑉1_ the number of CV1 folds, 𝑟_𝐶𝑉2_ the number of CV2 repetitions, 𝑘_𝐶𝑉2_ the number of CV2 folds, 𝐰̂^𝑖^_j_ the 𝑗th element of 𝑖th normalized weight vector 𝐰̂^𝑖^ = 𝐰^𝑖^/‖𝐰^𝑖^‖ in the SVM ensemble, 𝜎𝐰̂^𝑖^_j_ the standard deviation of 𝐰̂^𝑖^_j_. Akin to *Z*-scores, the CVR vectors or images were thresholded at CVR value ranges corresponding to an alpha level of 0.01 (-1.6 ~ 1.2) to delineate stable pattern elements across the cross-validation experiment.

**4.2. Sign-based consistency**

Furthermore, we implemented a sign-consistency-based method to statistically probe the relevance of the variables in our SVM ensembles. To this end, we adopted and extended the approach proposed by (Gómez-Verdejo et al., 2019) toward wrapper-based feature selection strategies. The variable importance of the 𝑗th pattern element in the SVM model ensembles as:


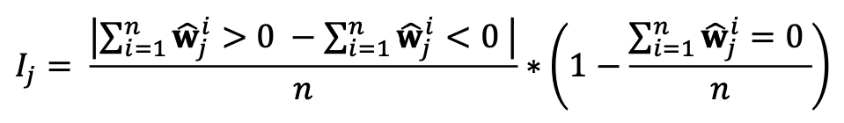


The first part of the equation measures the consistency of the weights assigned by the SVM ensemble to given pattern element. The importance 𝐼*_j_* is reduced by the second part of the equation, which measures the fraction of SVMs that de-selected the given pattern element during the wrapper-based optimization process. Hence, 𝐼*_j_* = 1, when the weights of 𝑗th pattern element all share the same sign and the element has been selected by all classifiers in the ensemble, or 𝐼*_j_* = 0, when positive and negative weights occur equally across the ensemble or given pattern element has been omitted by all classifiers in the ensemble. We defined a hypothesis test for 𝐼*_j_* , with:


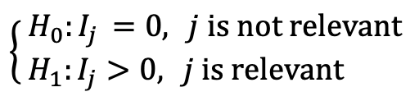


Significance thresholds for this hypothesis test were derived using the *Z*-statistic, defined as:


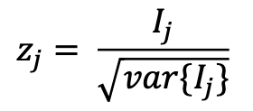


We used the normal cumulative distribution function to pick the right-tailed *P* value corresponding to the respective *Z*-score of the variable importance of 𝐼*_j_* . The *P* values were corrected for multiple comparisons using the false-discovery rate and statistical significance was defined at α=0.05.

***Table S15.*** *RESIS active treated group clinical + PRS model predictive pattern. Feature weights are extracted from the SVM model ensembles, spearman correlations are calculated between target and feature in univariate analysis, CV-Ratio method is described in supplement C4.1, SignConst method is described in supplement C4.2. All P-values are obtained from permutation analysis of P-CV predictors trained on respective original label distributions vs 1000 respective predictors trained on random label permutations, FDR-corrected for multiple tests.*

***Abbreviations:*** *CV-Ratio (mean): Mean Cross Validation Ratio, Feature weight (mean StErr): Standard error of mean feature weights, Spearman (mean): Mean Spearman coefficients, SignConst -log10 P (fdr): FDR-corrected -log10 P-values from Sign-based Consistency method, Feature selection prob: The percentage of classifiers in the ensemble which used this feature, PRS SZ: Schizophrenia PRS scores, PRS EA: Education Attainment PRS scores.*

| **Feature** | **CV-Ratio (mean)** | **Feature weight (mean)** | **Feature weight (mean StErr)** | **Spearman (mean)** | **SignConst**  **-log10 P (fdr)** | **Feature selection prob** |
| --- | --- | --- | --- | --- | --- | --- |
| ***Sociodemographic and clinical features*** | | | | | | |
| Marital status | -10.39 | -0.18 | 0.02 | -0.16 | 12.05 | 100.0% |
| Employment status | 10.74 | 0.25 | 0.02 | 0.15 | 12.05 | 100.0% |
| Housing status | -7.52 | -0.16 | 0.02 | -0.08 | 12.00 | 100.0% |
| Education (y) | 4.11 | 0.07 | 0.02 | 0.17 | 11.49 | 100.0% |
| Education parents | -9.79 | -0.24 | 0.02 | -0.17 | 12.05 | 100.0% |
| Alcohol abuse | 9.77 | 0.16 | 0.02 | 0.24 | 12.00 | 99.5% |
| Alcohol addiction | 3.37 | 0.04 | 0.01 | 0.20 | 8.21 | 89.7% |
| Substance abuse | 9.84 | 0.15 | 0.01 | 0.26 | 12.05 | 100.0% |
| Substance addiction | 8.99 | 0.19 | 0.02 | 0.33 | 12.05 | 100.0% |
| PANSS-PS | 4.98 | 0.10 | 0.02 | 0.05 | 11.75 | 100.0% |
| PANSS-NS | 2.97 | 0.06 | 0.02 | 0.07 | 7.93 | 100.0% |
| PANSS-GS | 1.67 | 0.02 | 0.01 | -0.01 | 4.30 | 100.0% |
| CDSS | -3.38 | -0.09 | 0.03 | -0.01 | 9.61 | 100.0% |
| CGI-S1 | -3.19 | -0.07 | 0.02 | 0.00 | 7.25 | 100.0% |
| GAF | -10.38 | -0.20 | 0.02 | -0.26 | 12.05 | 100.0% |
| MADRS-1 | 17.63 | 0.36 | 0.02 | 0.37 | 12.05 | 100.0% |
| MADRS-2 | 4.02 | 0.07 | 0.02 | 0.03 | 9.66 | 100.0% |
| MADRS-3 | 5.97 | 0.13 | 0.02 | 0.15 | 12.05 | 100.0% |
| MADRS-4 | 8.36 | 0.19 | 0.02 | 0.21 | 12.05 | 100.0% |
| MADRS-5 | 6.21 | 0.11 | 0.02 | 0.10 | 11.93 | 100.0% |
| MADRS-6 | -3.62 | -0.08 | 0.02 | -0.07 | 9.81 | 100.0% |
| MADRS-7 | 2.26 | 0.05 | 0.02 | 0.12 | 4.12 | 100.0% |
| MADRS-8 | 17.58 | 0.35 | 0.02 | 0.36 | 12.05 | 100.0% |
| MADRS-9 | -5.63 | -0.10 | 0.02 | -0.01 | 11.91 | 100.0% |
| MADRS-10 | 4.50 | 0.08 | 0.02 | 0.12 | 11.72 | 100.0% |
| ***Polygenic Risk Score (PRS) features*** | | | | | | |
| PRS SZ (phi:1e-1) | -4.69 | -0.08 | 0.02 | -0.12 | 5.64 | 100.0% |
| PRS SZ (phi:1e-2) | -5.43 | -0.08 | 0.01 | -0.13 | 5.69 | 100.0% |
| PRS SZ (phi:1e-3) | -4.90 | -0.07 | 0.01 | -0.11 | 6.53 | 100.0% |
| PRS SZ (phi:1e-4) | -2.02 | -0.03 | 0.02 | -0.13 | 4.00 | 100.0% |
| PRS SZ (phi:1e-5) | -0.81 | -0.01 | 0.02 | -0.09 | 1.59 | 100.0% |
| PRS SZ (phi:1e-6) | -2.02 | -0.04 | 0.02 | -0.15 | 5.16 | 100.0% |
| PRS EA (phi:1e-1) | -3.34 | -0.08 | 0.02 | -0.17 | 4.30 | 100.0% |
| PRS EA (phi:1e-2) | -6.09 | -0.12 | 0.02 | -0.23 | 6.78 | 100.0% |
| PRS EA (phi:1e-3) | -10.69 | -0.19 | 0.02 | -0.28 | 6.79 | 100.0% |
| PRS EA (phi:1e-4) | -11.34 | -0.22 | 0.02 | -0.32 | 6.79 | 100.0% |
| PRS EA (phi:1e-5) | -11.84 | -0.26 | 0.02 | -0.36 | 6.79 | 100.0% |
| PRS EA (phi:1e-6) | -11.25 | -0.19 | 0.02 | -0.29 | 6.79 | 100.0% |

**4.3 Mapping neuroanatomical predictive pattern to AAL and Yeo brain atlas**

We have extracted the neuroanatomical prediction pattern from sMRI models using the same technique as described in our previous work. The reliability of the gray matter density (GMD) pattern elements was measured in terms of a Cross-Validation Ratio (CVR) map [CVR = mean(**w**) / standard error(**w**)], where **w** are the weight vectors of the 5054 Support Vector Machine (SVM) models generated in the study’s repeated nested cross-validation setup]. The CVR map was thresholded at CVR ranges corresponding to an alpha level of 0.01. Reliable areas of GMD reduction in non-responders (NON-RESP) vs responders (RESP) are shaded in red colours, whereas areas of GMD increments are painted in green. The open-source 3D rendering software MRIcroGL (Rorden & Brett, 2000) available at https://www.nitrc.org/projects/mricrogl/ was used to overlay the CVR map on the MNI single-subject template.

**4.4 Neuroanatomical predictive pattern comparison between our previous work and current study**

We have extracted the neuroanatomical predictive patterns from our previous work (Koutsouleris et al., 2018) and compared it with the new retrained sMRI model used in the current study. We have conducted Wilcoxon signed rank test to assess whether there is a significant difference between the neuroanatomical predictive patterns between our previous work and current study, and no significant differences were found (p^non-responder CVR^ = 0.18, p^responder CVR^ = 0.91). These results indicate that the neuroanatomical predictive pattern extracted from the retrained sMRI model in our current study is statistically indifferent to our previous work, validating the replicability of our machine learning pipeline [Supplement S16, S17].

***Figure S16.*** *Reliability of the baseline Gray Matter Density predictive pattern extracted from sMRI model of our previous work. Red colour ranges indicating significant GMD increase voxels which are predicting responders to rTMS treatment in RESIS active group. Detailed figure explanation can be found in Figure 3-A of the main article.*


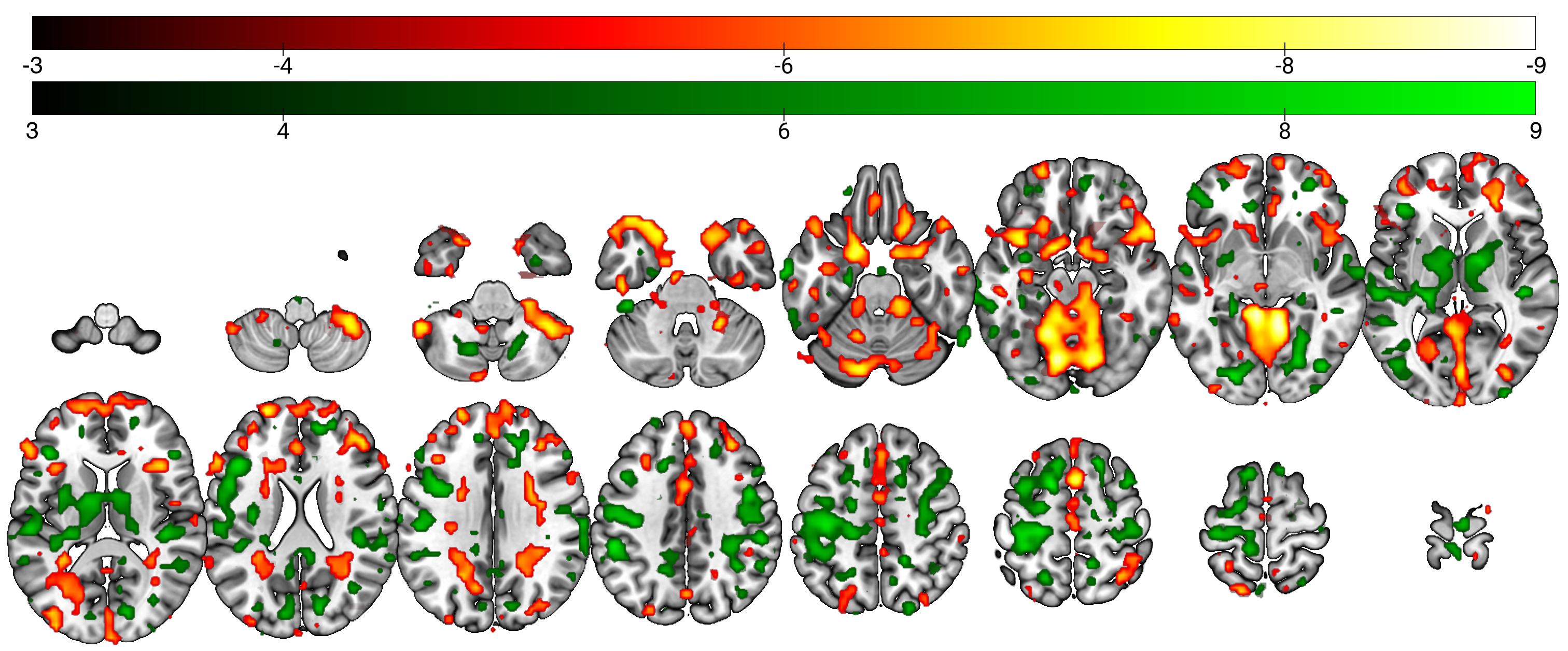


***Figure S17.*** *Reliability of the baseline Gray Matter Density predictive pattern extracted from sMRI model of the current study. Red colour ranges indicating significant GMD increase voxels which are predicting responders to rTMS treatment in RESIS active group. Detailed figure explanation can be found in Figure 3-A of the main article.*


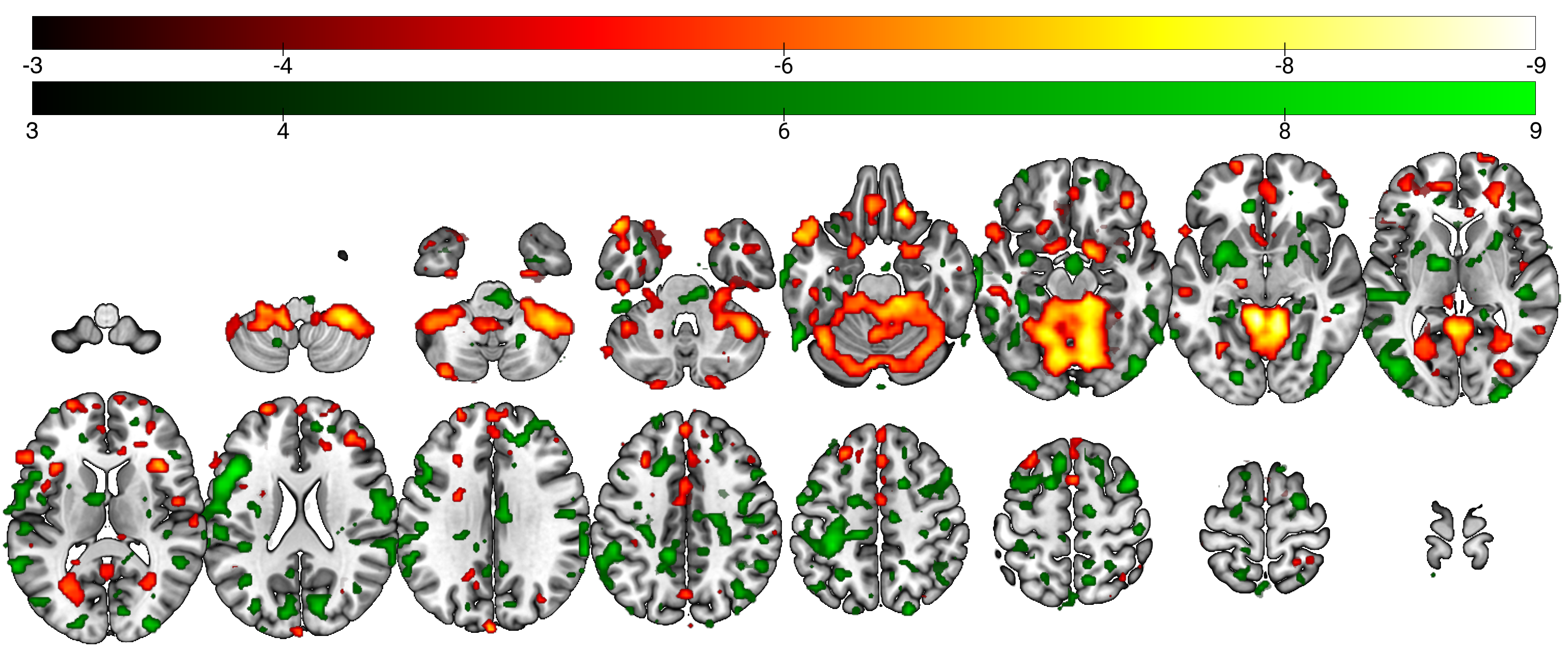


***Table S18.*** *Significant CVR voxels summed per AAL atlas ROI for predicting responder to rTMS treatment extracted from the sMRI model of our previous work. Only ROIs with more than 10% of significant voxels are listed.*

| **AAL ROI** | **ROI voxel count** | **ROI volume (mm3)** | **Significant voxel count** | **Significant volume** | **% significant voxels in ROI** |
| --- | --- | --- | --- | --- | --- |
| **Vermis** | 1511 | 12088 | 1178 | 9424 | 67.580794 |
| **Amygdala** | 468 | 3744 | 253 | 2024 | 53.753666 |
| **Olfactory** | 569 | 4552 | 253 | 2024 | 44.616906 |
| **OFCpost** | 1128 | 9024 | 486 | 3888 | 43.053316 |
| **OFClat** | 188 | 1504 | 80 | 640 | 42.553191 |
| **Temporal** | 7765 | 62120 | 2695 | 21560 | 42.513101 |
| **Cerebelum** | 21422 | 171376 | 6531 | 52248 | 40.802015 |
| **Lingual** | 4395 | 35160 | 1614 | 12912 | 36.899346 |
| **OFCmed** | 1171 | 9368 | 363 | 2904 | 30.359977 |
| **Supp** | 4518 | 36144 | 1293 | 10344 | 28.494666 |
| **Rectus** | 1597 | 12776 | 452 | 3616 | 27.688975 |
| **ParaHippocampal** | 2110 | 16880 | 561 | 4488 | 26.865692 |
| **Caudate** | 962 | 7696 | 254 | 2032 | 26.403326 |
| **Frontal** | 32304 | 258432 | 7920 | 63360 | 25.688182 |
| **Insula** | 3628 | 29024 | 820 | 6560 | 22.607263 |
| **Calcarine** | 4119 | 32952 | 964 | 7712 | 22.27691 |
| **Hippocampus** | 932 | 7456 | 200 | 1600 | 21.459227 |
| **Parietal** | 5632 | 45056 | 1223 | 9784 | 20.878794 |
| **Cingulate** | 6857 | 54856 | 1238 | 9904 | 17.804201 |
| **Fusiform** | 4828 | 38624 | 846 | 6768 | 17.592915 |
| **OFCant** | 443 | 3544 | 74 | 592 | 16.704289 |
| **Occipital** | 6781 | 54248 | 1093 | 8744 | 15.166394 |
| **Precuneus** | 6793 | 54344 | 818 | 6544 | 12.099841 |
| **Cuneus** | 1526 | 12208 | 181 | 1448 | 11.861075 |

***Table S19.*** *Significant CVR voxels summed per AAL atlas ROI for predicting responder to rTMS treatment extracted from the sMRI model of the current study. Only ROIs with more than 10% of significant voxels are listed.*

| **AAL ROI** | **ROI voxel count** | **ROI volume (mm3)** | **Significant voxel count** | **Significant volume** | **% significant voxels in ROI** |
| --- | --- | --- | --- | --- | --- |
| **Vermis** | 1623 | 12984 | 1371 | 10968 | 89.157104 |
| **Cerebelum** | 22375 | 179000 | 9771 | 78168 | 58.12904 |
| **Olfactory** | 569 | 4552 | 270 | 2160 | 47.491349 |
| **Rectus** | 1597 | 12776 | 579 | 4632 | 35.201027 |
| **Lingual** | 4395 | 35160 | 1504 | 12032 | 34.254903 |
| **OFCmed** | 1171 | 9368 | 374 | 2992 | 31.630215 |
| **Amygdala** | 468 | 3744 | 143 | 1144 | 29.52346 |
| **Fusiform** | 4828 | 38624 | 1407 | 11256 | 29.179707 |
| **Temporal** | 7765 | 62120 | 1816 | 14528 | 28.776397 |
| **ParaHippocampal** | 2110 | 16880 | 560 | 4480 | 26.501586 |
| **OFClat** | 188 | 1504 | 40 | 320 | 21.276596 |
| **OFCpost** | 1128 | 9024 | 225 | 1800 | 19.925209 |
| **Calcarine** | 4119 | 32952 | 660 | 5280 | 15.847611 |
| **Frontal** | 24510 | 196080 | 4376 | 35008 | 15.370367 |
| **OFCant** | 648 | 5184 | 94 | 752 | 14.506173 |
| **Cingulate** | 6857 | 54856 | 988 | 7904 | 14.413538 |
| **Cuneus** | 1526 | 12208 | 218 | 1744 | 14.285714 |
| **Caudate** | 962 | 7696 | 128 | 1024 | 13.305613 |
| **Supp** | 4518 | 36144 | 586 | 4688 | 12.857092 |

***Table S20.*** *Significant CVR voxels summed per AAL atlas ROI for predicting non-responder to rTMS treatment extracted from the sMRI model of our previous work. Only ROIs with more than 10% of significant voxels are listed.*

| **AAL ROI** | **ROI voxel count** | **ROI volume (mm3)** | **Significant voxel count** | **Significant volume** | **% significant voxels in ROI** |
| --- | --- | --- | --- | --- | --- |
| **Pallidum** | 573 | 4584 | 361 | 2888 | 62.943077 |
| **Thalamus** | 2157 | 17256 | 1201 | 9608 | 55.661564 |
| **Heschl** | 474 | 3792 | 237 | 1896 | 50.203481 |
| **Precentral** | 6907 | 55256 | 2897 | 23176 | 41.803635 |
| **Postcentral** | 7715 | 61720 | 3168 | 25344 | 40.894103 |
| **SupraMarginal** | 3230 | 25840 | 1220 | 9760 | 37.721631 |
| **ParaHippocampal** | 978 | 7824 | 350 | 2800 | 35.787321 |
| **Cingulate** | 2111 | 16888 | 486 | 3888 | 31.248108 |
| **OFCant** | 1091 | 8728 | 305 | 2440 | 28.604249 |
| **Putamen** | 2073 | 16584 | 589 | 4712 | 28.575108 |
| **Cuneus** | 2950 | 23600 | 757 | 6056 | 25.744538 |
| **Paracentral** | 2185 | 17480 | 592 | 4736 | 25.1491 |
| **Temporal** | 17136 | 137088 | 3999 | 31992 | 24.325933 |
| **Parietal** | 8079 | 64632 | 1794 | 14352 | 22.178581 |
| **Occipital** | 7298 | 58384 | 1444 | 11552 | 21.898214 |
| **Frontal** | 26620 | 212960 | 5595 | 44760 | 21.761713 |
| **Supp** | 4518 | 36144 | 980 | 7840 | 21.748865 |
| **Lingual** | 4395 | 35160 | 947 | 7576 | 21.603767 |
| **Rolandic** | 2321 | 18568 | 479 | 3832 | 21.228817 |
| **Hippocampus** | 932 | 7456 | 191 | 1528 | 20.493562 |
| **Calcarine** | 1861 | 14888 | 351 | 2808 | 18.860828 |
| **Angular** | 1752 | 14016 | 278 | 2224 | 15.86758 |
| **Precuneus** | 6793 | 54344 | 1044 | 8352 | 15.338178 |
| **Insula** | 1858 | 14864 | 258 | 2064 | 13.885899 |
| **Cerebelum** | 4780 | 38240 | 654 | 5232 | 13.345861 |
| **Fusiform** | 4828 | 38624 | 603 | 4824 | 12.563826 |
| **Amygdala** | 220 | 1760 | 22 | 176 | 10 |

***Table S21.*** *Significant CVR voxels summed per AAL atlas ROI for predicting non-responder to rTMS treatment extracted from the sMRI model of the current study. Only ROIs with more than 10% of significant voxels are listed.*

| **AAL ROI** | **ROI voxel count** | **ROI volume (mm3)** | **Significant voxel count** | **Significant volume** | **% significant voxels in ROI** |
| --- | --- | --- | --- | --- | --- |
| **Pallidum** | 573 | 4584 | 325 | 2600 | 56.538274 |
| **SupraMarginal** | 3230 | 25840 | 1164 | 9312 | 38.489617 |
| **Putamen** | 2073 | 16584 | 722 | 5776 | 34.868654 |
| **Occipital** | 10077 | 80616 | 2796 | 22368 | 29.754441 |
| **Postcentral** | 7715 | 61720 | 2246 | 17968 | 29.068538 |
| **ParaHippocampal** | 978 | 7824 | 277 | 2216 | 28.323108 |
| **Cuneus** | 2950 | 23600 | 780 | 6240 | 26.617838 |
| **Amygdala** | 220 | 1760 | 52 | 416 | 23.636364 |
| **OFCant** | 648 | 5184 | 150 | 1200 | 23.148148 |
| **Rolandic** | 2321 | 18568 | 499 | 3992 | 22.523583 |
| **Parietal** | 8079 | 64632 | 1748 | 13984 | 22.498045 |
| **Frontal** | 25884 | 207072 | 5196 | 41568 | 22.25295 |
| **Precentral** | 6907 | 55256 | 1481 | 11848 | 21.34109 |
| **Angular** | 2925 | 23400 | 585 | 4680 | 18.765985 |
| **Precuneus** | 6793 | 54344 | 1256 | 10048 | 18.588158 |
| **Heschl** | 474 | 3792 | 86 | 688 | 18.168675 |
| **Temporal** | 22300 | 178400 | 4137 | 33096 | 17.932903 |
| **Lingual** | 4395 | 35160 | 766 | 6128 | 17.645585 |
| **Fusiform** | 2310 | 18480 | 399 | 3192 | 17.272727 |
| **Calcarine** | 1861 | 14888 | 281 | 2248 | 15.099409 |
| **Cingulate** | 4942 | 39536 | 807 | 6456 | 14.996993 |
| **Hippocampus** | 932 | 7456 | 138 | 1104 | 14.806867 |
| **Supp** | 4518 | 36144 | 650 | 5200 | 14.422349 |

***Table S22.*** *Significant CVR voxels summed by brain networks from Yeo atlas for predicting responder to rTMS treatment extracted from the sMRI model of the current study.*

| **Brain network** | **Network voxel count** | **Network volume (mm3)** | **Significant voxel count** | **Significant volume** | **% significant voxels in network** |
| --- | --- | --- | --- | --- | --- |
| **Default** | 14562 | 116496 | 3264 | 26112 | 25.586899 |
| **Limbic** | 9055 | 72440 | 2141 | 17128 | 25.558093 |
| **Frontalparietal** | 3513 | 28104 | 582 | 4656 | 23.034347 |
| **Visual** | 20355 | 162840 | 3301 | 26408 | 16.194587 |
| **Ventral attention** | 5725 | 45800 | 744 | 5952 | 13.14125 |

***Table S23.*** *Significant CVR voxels summed by brain networks from Yeo atlas for predicting non-responder to rTMS treatment extracted from the sMRI model of the current study.*

| **Brain network** | **Network voxel count** | **Network volume (mm3)** | **Significant voxel count** | **Significant volume** | **% significant voxels in network** |
| --- | --- | --- | --- | --- | --- |
| **Ventral attention** | 4115 | 32920 | 1266 | 10128 | 29.177318 |
| **Frontalparietal** | 11362 | 90896 | 2267 | 18136 | 27.506867 |
| **Dorsal attention** | 10028 | 80224 | 2011 | 16088 | 26.630622 |
| **Visual** | 20355 | 162840 | 4246 | 33968 | 20.942096 |
| **Default** | 20273 | 162184 | 3526 | 28208 | 19.438797 |
| **Somatomotor** | 16261 | 130088 | 3132 | 25056 | 19.213609 |
| **Limbic** | 2844 | 22752 | 401 | 3208 | 14.099859 |

#### **C5. RESIS active post-hoc cross-modalities correlation analyses**

We implemented a series of post-hoc correlation analyses to assess the correlation between clinical, PRS data and sMRI images in order to find potential cross-modalities pattern which could form a bridge linking the predictive patterns identified by the sMRI model and the clinical+PRS model. First we correct for covariate effects for all modalities (sMRI, clinical, PRS) following the same preprocessing pipeline described in supplement 3.1 and 3.2. The only alteration is that for the correlation analyses we used ComBat technique (Behdenna et al., 2020; Johnson et al., 2007) to correct for site effects. We further processed sMRI imaging data by transforming the voxel-based GMD map into Region of Interest (ROI) based GMD map using the AAL atlas and brain network-based GMD map using the Yeo atlas(Thomas Yeo et al., 2011). These two atlases were used for Neuroanatomical predictive patterns extraction from the sMRI model [supplement S18-S23].

After the preprocessing, we conducted univariate Pearson correlation analyses between each clinical and PRS feature used in our clinical+PRS model [supplement S15] and GMD organised in ROIs and brain networks. Only significant correlations (p<0.05) are listed in the tables below [supplement S25-S32]. All analyses in this chapter are done in Python using neuroimaging, statistical and machine learning packages (nilearn, NiBabel, pandas, numpy, scipy, scikit-learn).

***Figure S24. Left:*** *scatter plot of case-wise prediction decision scores between the sMRI model and clinical+PRS model with R^2^ value between the two models.* ***Right:*** *scatter plot of case-wise predicted treatment response probabilities between the sMRI model and clinical+PRS model with R^2^ value between the two models.*


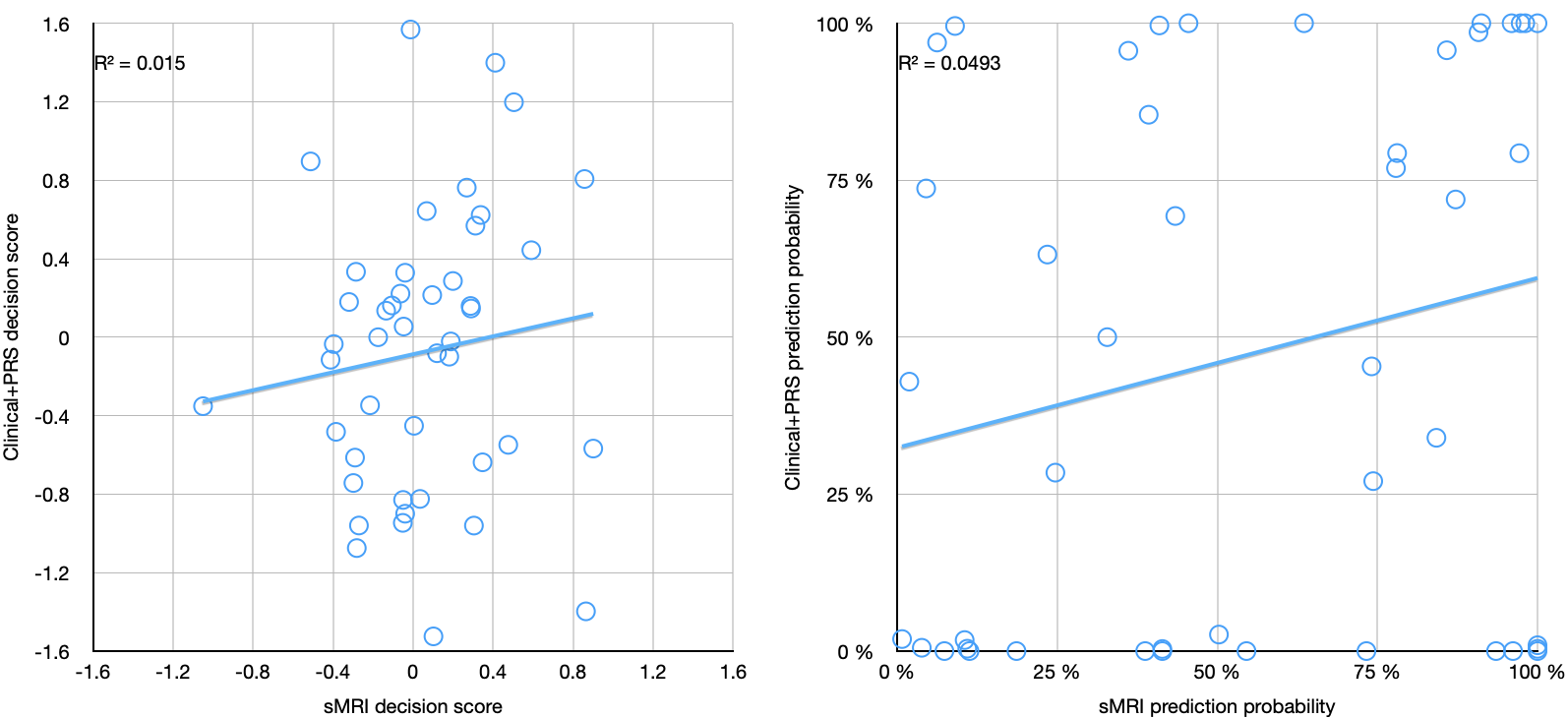


***Table S25.*** *Significant correlations between clinical features and ROIs according to AAL atlas from the GMD map, ranked alphabetically.*

***Column definition:*** *AAL ROI: detailed Region of Interest defined by AAL atlas, r (abs): absolute r-value from Pearson correlation, r: r-value from Pearson correlation, P: p-value from Pearson correlation, P (fdr): p-value from Pearson correlation FDR-corrected for multiple testing. Clinical feature: name of the clinical feature used in the clinical+PRS model, Feature weight rank: the rank of the clinical feature according to mean feature weights in the clinical+PRS model, see supplement S15 for detail.*

| **AAL ROI** | **r (abs)** | **r** | **P** | **P (fdr)** | **Clinical feature** | **Feature weight rank** |
| --- | --- | --- | --- | --- | --- | --- |
| Amygdala_R | 0.341 | -0.341 | 0.022 | 0.047 | GAF | 9 |
| Angular_L | 0.350 | -0.350 | 0.018 | 0.047 | MADRS-7 | 28 |
| Angular_L | 0.303 | -0.303 | 0.043 | 0.049 | MADRS-6 | 17 |
| Angular_R | 0.312 | 0.312 | 0.037 | 0.049 | MADRS-8 | 1 |
| Angular_R | 0.312 | 0.312 | 0.037 | 0.049 | MADRS-7 | 28 |
| Calcarine_R | 0.516 | 0.516 | 0.000 | 0.016 | PANSS-PS | 19 |
| Calcarine_R | 0.347 | 0.347 | 0.020 | 0.047 | Alcohol addiction | 31 |
| Calcarine_R | 0.340 | 0.340 | 0.022 | 0.047 | GAF | 9 |
| Calcarine_R | 0.306 | 0.306 | 0.041 | 0.049 | MADRS-9 | 26 |
| Caudate_L | 0.313 | 0.313 | 0.037 | 0.049 | CDSS | 22 |
| Caudate_L | 0.309 | -0.309 | 0.039 | 0.049 | GAF | 9 |
| Caudate_R | 0.346 | -0.346 | 0.020 | 0.047 | GAF | 9 |
| Caudate_R | 0.307 | -0.307 | 0.040 | 0.049 | MADRS-3 | 23 |
| Cerebelum_3_L | 0.379 | -0.379 | 0.010 | 0.039 | MADRS-5 | 21 |
| Cerebelum_4_5_L | 0.304 | -0.304 | 0.042 | 0.049 | GAF | 9 |
| Cerebelum_4_5_R | 0.304 | -0.304 | 0.042 | 0.049 | PANSS-GS | 37 |
| Cerebelum_6_R | 0.296 | 0.296 | 0.048 | 0.049 | GAF | 9 |
| Cerebelum_7b_L | 0.323 | -0.323 | 0.030 | 0.049 | MADRS-1 | 2 |
| Cerebelum_7b_L | 0.303 | -0.303 | 0.043 | 0.049 | CGI-S1 | 36 |
| Cerebelum_8_L | 0.303 | -0.303 | 0.043 | 0.049 | MADRS-4 | 6 |
| Cerebelum_9_L | 0.420 | -0.420 | 0.004 | 0.028 | GAF | 9 |
| Cerebelum_9_L | 0.390 | -0.390 | 0.008 | 0.036 | Substance addiction | 15 |
| Cerebelum_9_L | 0.383 | -0.383 | 0.009 | 0.038 | Substance abuse | 16 |
| Cerebelum_9_L | 0.330 | 0.330 | 0.027 | 0.049 | MADRS-4 | 6 |
| Cerebelum_9_L | 0.317 | -0.317 | 0.034 | 0.049 | PANSS-PS | 19 |
| Cerebelum_Crus1_L | 0.444 | -0.444 | 0.002 | 0.028 | PANSS-PS | 19 |
| Cerebelum_Crus1_L | 0.344 | -0.344 | 0.021 | 0.047 | MADRS-10 | 27 |
| Cerebelum_Crus1_L | 0.306 | -0.306 | 0.041 | 0.049 | Alcohol abuse | 13 |
| Cerebelum_Crus1_R | 0.397 | -0.397 | 0.007 | 0.034 | MADRS-6 | 17 |
| Cerebelum_Crus2_R | 0.324 | -0.324 | 0.030 | 0.049 | MADRS-7 | 28 |
| Cingulum_Ant_L | 0.333 | 0.333 | 0.026 | 0.049 | MADRS-5 | 21 |
| Cingulum_Ant_R | 0.513 | -0.513 | 0.000 | 0.016 | MADRS-10 | 27 |
| Cingulum_Ant_R | 0.469 | -0.469 | 0.001 | 0.025 | MADRS-2 | 33 |
| Cingulum_Ant_R | 0.433 | -0.433 | 0.003 | 0.028 | PANSS-GS | 37 |
| Cingulum_Ant_R | 0.418 | -0.418 | 0.004 | 0.028 | CDSS | 22 |
| Cingulum_Ant_R | 0.368 | -0.368 | 0.013 | 0.043 | MADRS-6 | 17 |
| Cingulum_Ant_R | 0.365 | -0.365 | 0.014 | 0.043 | PANSS-PS | 19 |
| Cingulum_Ant_R | 0.331 | -0.331 | 0.026 | 0.049 | MADRS-1 | 2 |
| Cingulum_Ant_R | 0.303 | -0.303 | 0.043 | 0.049 | MADRS-9 | 26 |
| Cingulum_Mid_L | 0.377 | 0.377 | 0.011 | 0.039 | MADRS-4 | 6 |
| Cingulum_Mid_R | 0.300 | -0.300 | 0.046 | 0.049 | PANSS-NS | 29 |
| Cingulum_Post_R | 0.337 | 0.337 | 0.024 | 0.048 | MADRS-8 | 1 |
| Cingulum_Post_R | 0.303 | 0.303 | 0.043 | 0.049 | MADRS-2 | 33 |
| Cuneus_L | 0.321 | -0.321 | 0.031 | 0.049 | MADRS-6 | 17 |
| Frontal_Inf_Oper_R | 0.326 | 0.326 | 0.029 | 0.049 | MADRS-3 | 23 |
| Frontal_Inf_Oper_R | 0.297 | 0.297 | 0.048 | 0.049 | MADRS-2 | 33 |
| Frontal_Inf_Tri_L | 0.345 | 0.345 | 0.020 | 0.047 | PANSS-PS | 19 |
| Frontal_Inf_Tri_L | 0.333 | 0.333 | 0.025 | 0.049 | Substance addiction | 15 |
| Frontal_Inf_Tri_R | 0.343 | -0.343 | 0.021 | 0.047 | MADRS-7 | 28 |
| Frontal_Inf_Tri_R | 0.299 | 0.299 | 0.046 | 0.049 | PANSS-PS | 19 |
| Frontal_Med_Orb_L | 0.362 | 0.362 | 0.015 | 0.044 | MADRS-1 | 2 |
| Frontal_Med_Orb_L | 0.346 | 0.346 | 0.020 | 0.047 | Substance addiction | 15 |
| Frontal_Med_Orb_L | 0.333 | 0.333 | 0.025 | 0.049 | MADRS-10 | 27 |
| Frontal_Med_Orb_L | 0.308 | 0.308 | 0.039 | 0.049 | MADRS-9 | 26 |
| Frontal_Med_Orb_L | 0.298 | 0.298 | 0.047 | 0.049 | Alcohol abuse | 13 |
| Frontal_Mid_L | 0.441 | 0.441 | 0.002 | 0.028 | MADRS-5 | 21 |
| Frontal_Mid_L | 0.384 | 0.384 | 0.009 | 0.037 | Substance abuse | 16 |
| Frontal_Mid_L | 0.311 | 0.311 | 0.038 | 0.049 | Substance addiction | 15 |
| Frontal_Mid_Orb_R | 0.299 | 0.299 | 0.046 | 0.049 | MADRS-8 | 1 |
| Frontal_Mid_R | 0.351 | -0.351 | 0.018 | 0.047 | MADRS-7 | 28 |
| Frontal_Sup_L | 0.337 | 0.337 | 0.023 | 0.048 | MADRS-10 | 27 |
| Frontal_Sup_Medial_R | 0.326 | -0.326 | 0.029 | 0.049 | Alcohol addiction | 31 |
| Frontal_Sup_Medial_R | 0.319 | -0.319 | 0.033 | 0.049 | Substance addiction | 15 |
| Frontal_Sup_Medial_R | 0.295 | -0.295 | 0.049 | 0.049 | PANSS-PS | 19 |
| Frontal_Sup_Orb_L | 0.425 | -0.425 | 0.004 | 0.028 | MADRS-2 | 33 |
| Frontal_Sup_Orb_R | 0.327 | 0.327 | 0.028 | 0.049 | MADRS-5 | 21 |
| Frontal_Sup_Orb_R | 0.305 | 0.305 | 0.042 | 0.049 | MADRS-6 | 17 |
| Fusiform_L | 0.311 | 0.311 | 0.037 | 0.049 | MADRS-3 | 23 |
| Fusiform_R | 0.318 | -0.318 | 0.033 | 0.049 | MADRS-5 | 21 |
| Heschl_R | 0.411 | 0.411 | 0.005 | 0.030 | CGI-S1 | 36 |
| Hippocampus_R | 0.533 | 0.533 | 0.000 | 0.016 | MADRS-10 | 27 |
| Hippocampus_R | 0.495 | 0.495 | 0.001 | 0.016 | Substance addiction | 15 |
| Hippocampus_R | 0.480 | 0.480 | 0.001 | 0.021 | PANSS-GS | 37 |
| Hippocampus_R | 0.441 | 0.441 | 0.002 | 0.028 | MADRS-9 | 26 |
| Hippocampus_R | 0.425 | 0.425 | 0.004 | 0.028 | MADRS-2 | 33 |
| Hippocampus_R | 0.422 | -0.422 | 0.004 | 0.028 | MADRS-4 | 6 |
| Hippocampus_R | 0.399 | 0.399 | 0.007 | 0.034 | Substance abuse | 16 |
| Hippocampus_R | 0.368 | 0.368 | 0.013 | 0.043 | CDSS | 22 |
| Hippocampus_R | 0.364 | 0.364 | 0.014 | 0.043 | PANSS-PS | 19 |
| Hippocampus_R | 0.358 | 0.358 | 0.016 | 0.045 | MADRS-6 | 17 |
| Hippocampus_R | 0.345 | 0.345 | 0.020 | 0.047 | MADRS-3 | 23 |
| Hippocampus_R | 0.305 | -0.305 | 0.042 | 0.049 | MADRS-5 | 21 |
| Insula_L | 0.321 | -0.321 | 0.032 | 0.049 | MADRS-7 | 28 |
| Lingual_L | 0.438 | -0.438 | 0.003 | 0.028 | PANSS-PS | 19 |
| Lingual_R | 0.377 | 0.377 | 0.011 | 0.039 | PANSS-PS | 19 |
| Occipital_Inf_L | 0.359 | -0.359 | 0.015 | 0.045 | Substance abuse | 16 |
| Occipital_Inf_L | 0.325 | -0.325 | 0.029 | 0.049 | PANSS-PS | 19 |
| Occipital_Mid_L | 0.326 | 0.326 | 0.029 | 0.049 | MADRS-8 | 1 |
| Occipital_Mid_L | 0.317 | 0.317 | 0.034 | 0.049 | CGI-S1 | 36 |
| Occipital_Mid_L | 0.302 | 0.302 | 0.044 | 0.049 | MADRS-9 | 26 |
| Occipital_Mid_R | 0.295 | 0.295 | 0.049 | 0.049 | MADRS-3 | 23 |
| Occipital_Sup_L | 0.318 | 0.318 | 0.033 | 0.049 | CDSS | 22 |
| Occipital_Sup_R | 0.329 | -0.329 | 0.027 | 0.049 | GAF | 9 |
| Pallidum_L | 0.459 | -0.459 | 0.002 | 0.025 | Alcohol addiction | 31 |
| Pallidum_L | 0.433 | -0.433 | 0.003 | 0.028 | Substance addiction | 15 |
| Pallidum_L | 0.392 | -0.392 | 0.008 | 0.036 | PANSS-GS | 37 |
| Pallidum_L | 0.376 | -0.376 | 0.011 | 0.039 | MADRS-10 | 27 |
| Pallidum_L | 0.329 | -0.329 | 0.027 | 0.049 | PANSS-PS | 19 |
| Paracentral_Lobule_R | 0.296 | -0.296 | 0.048 | 0.049 | PANSS-NS | 29 |
| Parietal_Inf_L | 0.342 | 0.342 | 0.021 | 0.047 | MADRS-9 | 26 |
| Parietal_Inf_R | 0.325 | -0.325 | 0.029 | 0.049 | PANSS-GS | 37 |
| Parietal_Sup_L | 0.360 | -0.360 | 0.015 | 0.045 | PANSS-GS | 37 |
| Postcentral_R | 0.500 | -0.500 | 0.000 | 0.016 | PANSS-PS | 19 |
| Postcentral_R | 0.461 | -0.461 | 0.001 | 0.025 | MADRS-10 | 27 |
| Postcentral_R | 0.411 | -0.411 | 0.005 | 0.030 | MADRS-2 | 33 |
| Postcentral_R | 0.408 | -0.408 | 0.005 | 0.030 | Alcohol abuse | 13 |
| Postcentral_R | 0.404 | -0.404 | 0.006 | 0.032 | MADRS-8 | 1 |
| Postcentral_R | 0.384 | -0.384 | 0.009 | 0.037 | MADRS-1 | 2 |
| Postcentral_R | 0.337 | -0.337 | 0.024 | 0.048 | PANSS-GS | 37 |
| Postcentral_R | 0.331 | -0.331 | 0.026 | 0.049 | CDSS | 22 |
| Postcentral_R | 0.315 | -0.315 | 0.035 | 0.049 | Substance abuse | 16 |
| Postcentral_R | 0.296 | -0.296 | 0.048 | 0.049 | CGI-S1 | 36 |
| Precentral_L | 0.401 | 0.401 | 0.006 | 0.033 | MADRS-5 | 21 |
| Precentral_L | 0.313 | -0.313 | 0.036 | 0.049 | MADRS-2 | 33 |
| Precentral_L | 0.298 | -0.298 | 0.047 | 0.049 | CGI-S1 | 36 |
| Precentral_R | 0.310 | 0.310 | 0.038 | 0.049 | MADRS-2 | 33 |
| Precuneus_L | 0.315 | -0.315 | 0.035 | 0.049 | MADRS-9 | 26 |
| Precuneus_R | 0.328 | 0.328 | 0.028 | 0.049 | MADRS-1 | 2 |
| Precuneus_R | 0.299 | 0.299 | 0.046 | 0.049 | MADRS-2 | 33 |
| Putamen_L | 0.418 | 0.418 | 0.004 | 0.028 | PANSS-PS | 19 |
| Supp_Motor_Area_R | 0.386 | 0.386 | 0.009 | 0.037 | Substance abuse | 16 |
| SupraMarginal_R | 0.356 | -0.356 | 0.017 | 0.045 | MADRS-7 | 28 |
| SupraMarginal_R | 0.307 | -0.307 | 0.040 | 0.049 | PANSS-GS | 37 |
| SupraMarginal_R | 0.301 | -0.301 | 0.045 | 0.049 | Alcohol abuse | 13 |
| Temporal_Inf_L | 0.432 | 0.432 | 0.003 | 0.028 | PANSS-PS | 19 |
| Temporal_Inf_L | 0.324 | -0.324 | 0.030 | 0.049 | MADRS-4 | 6 |
| Temporal_Inf_L | 0.314 | 0.314 | 0.036 | 0.049 | MADRS-9 | 26 |
| Temporal_Inf_R | 0.389 | -0.389 | 0.008 | 0.036 | MADRS-1 | 2 |
| Temporal_Inf_R | 0.317 | -0.317 | 0.034 | 0.049 | Substance abuse | 16 |
| Temporal_Inf_R | 0.306 | -0.306 | 0.041 | 0.049 | Substance addiction | 15 |
| Temporal_Inf_R | 0.296 | -0.296 | 0.048 | 0.049 | MADRS-10 | 27 |
| Temporal_Mid_L | 0.425 | -0.425 | 0.004 | 0.028 | PANSS-NS | 29 |
| Temporal_Mid_L | 0.364 | -0.364 | 0.014 | 0.043 | Substance abuse | 16 |
| Temporal_Mid_L | 0.356 | -0.356 | 0.016 | 0.045 | CGI-S1 | 36 |
| Temporal_Mid_L | 0.332 | -0.332 | 0.026 | 0.049 | MADRS-3 | 23 |
| Temporal_Mid_L | 0.309 | -0.309 | 0.039 | 0.049 | MADRS-1 | 2 |
| Temporal_Mid_R | 0.368 | -0.368 | 0.013 | 0.043 | MADRS-5 | 21 |
| Temporal_Pole_Mid_L | 0.409 | -0.409 | 0.005 | 0.030 | CGI-S1 | 36 |
| Temporal_Pole_Mid_L | 0.370 | -0.370 | 0.012 | 0.043 | Substance abuse | 16 |
| Temporal_Pole_Sup_R | 0.302 | -0.302 | 0.044 | 0.049 | MADRS-5 | 21 |
| Temporal_Sup_L | 0.341 | 0.341 | 0.022 | 0.047 | MADRS-6 | 17 |
| Thalamus_L | 0.315 | 0.315 | 0.035 | 0.049 | Alcohol abuse | 13 |
| Thalamus_R | 0.342 | 0.342 | 0.022 | 0.047 | PANSS-PS | 19 |
| Thalamus_R | 0.306 | 0.306 | 0.041 | 0.049 | MADRS-10 | 27 |
| Thalamus_R | 0.304 | -0.304 | 0.042 | 0.049 | MADRS-4 | 6 |
| Thalamus_R | 0.302 | 0.302 | 0.044 | 0.049 | PANSS-GS | 37 |
| Vermis_3 | 0.351 | 0.351 | 0.018 | 0.047 | MADRS-5 | 21 |
| Vermis_4_5 | 0.343 | -0.343 | 0.021 | 0.047 | PANSS-GS | 37 |
| Vermis_4_5 | 0.307 | -0.307 | 0.040 | 0.049 | MADRS-6 | 17 |
| Vermis_9 | 0.342 | -0.342 | 0.021 | 0.047 | MADRS-10 | 27 |
| Vermis_9 | 0.300 | -0.300 | 0.046 | 0.049 | PANSS-PS | 19 |

***Table S26.*** *Significant correlations between clinical features and ROIs according to AAL atlas from the GMD map summed by AAL brain regions, ranked by ROI count.*

***Column definition:*** *AAL region: brain region defined by AAL atlas, ROI count: the number of AAL ROIs which contains a significant correlation belonging to the brain region. Mean P (fdr): Mean p-value from all ROIs belonging to the brain region.*

| **AAL region** | **ROI count** | **Mean P (fdr)** |
| --- | --- | --- |
| **Frontal** | 23 | 0.045 |
| **Cerebellum + Vermis** | 18 | 0.045 |
| **Temporal** | 17 | 0.044 |
| **Cingulum** | 13 | 0.044 |
| **Hippocampus** | 12 | 0.033 |
| **Postcentral** | 10 | 0.037 |
| **Occipital** | 8 | 0.048 |
| **Pallidum** | 5 | 0.036 |
| **Thalamus** | 5 | 0.049 |
| **Angular** | 4 | 0.048 |
| **Calcarine** | 4 | 0.040 |
| **Caudate** | 4 | 0.048 |
| **Precentral** | 4 | 0.046 |
| **Parietal** | 3 | 0.047 |
| **Precuneus** | 3 | 0.049 |
| **SupraMarginal** | 3 | 0.048 |
| **Fusiform** | 2 | 0.049 |
| **Lingual** | 2 | 0.034 |
| **Amygdala** | 1 | 0.047 |
| **Cuneus** | 1 | 0.049 |
| **Heschl** | 1 | 0.030 |
| **Insula** | 1 | 0.049 |
| **Paracentral** | 1 | 0.049 |
| **Putamen** | 1 | 0.028 |
| **Supp** | 1 | 0.037 |

***Table S27.*** *Significant correlations between clinical and sociodemographic features and ROIs according to AAL atlas from the GMD map summed by clinical features, ranked by Feature weight rank.*

***Column definition:*** *Clinical feature: name of the clinical and sociodemographic feature used in the clinical+PRS model, ROI count: the number of AAL ROIs which contains a significant correlation with the clinical feature. Mean P (fdr): Mean p-value from all ROIs with contains a significant correlation with the clinical feature. Feature weight rank: the rank of the clinical feature according to mean feature weights in the clinical+PRS model, see supplement S15 for detail.*

| **Clinical feature** | **ROI count** | **Mean P (fdr)** | **Feature weight rank** |
| --- | --- | --- | --- |
| **MADRS-8** | 5 | 0.045 | 1 |
| **MADRS-1** | 7 | 0.045 | 2 |
| **MADRS-4** | 6 | 0.044 | 6 |
| **GAF** | 8 | 0.046 | 9 |
| **Alcohol abuse** | 5 | 0.045 | 13 |
| **Substance addiction** | 8 | 0.040 | 15 |
| **Substance abuse** | 9 | 0.042 | 16 |
| **MADRS-6** | 8 | 0.045 | 17 |
| **PANSS-PS** | 17 | 0.039 | 19 |
| **MADRS-5** | 10 | 0.043 | 21 |
| **CDSS** | 5 | 0.044 | 22 |
| **MADRS-3** | 6 | 0.049 | 23 |
| **MADRS-9** | 8 | 0.046 | 26 |
| **MADRS-10** | 10 | 0.038 | 27 |
| **MADRS-7** | 7 | 0.047 | 28 |
| **PANSS-NS** | 3 | 0.042 | 29 |
| **Alcohol addiction** | 3 | 0.040 | 31 |
| **MADRS-2** | 9 | 0.040 | 33 |
| **CGI-S1** | 7 | 0.043 | 36 |
| **PANSS-GS** | 10 | 0.042 | 37 |

***Table S28.*** *Significant correlations between clinical features and brain networks according to Yeo atlas from the GMD map, ranked by Feature weight rank.*

***Column definition:*** *Brain network: 7 brain networks defined by Yeo atlas, refer to supplement S25 for the other column definitions.*

| **Brain network** | **r (abs)** | **r** | **P** | **P (fdr)** | **Clinical feature** | **Feature weight rank** |
| --- | --- | --- | --- | --- | --- | --- |
| **Limbic** | 0.302 | -0.302 | 0.044 | 0.046 | MADRS-1 | 2 |
| **Default** | 0.303 | 0.303 | 0.043 | 0.046 | Substance addiction | 15 |
| **Default** | 0.382 | 0.382 | 0.010 | 0.019 | Substance abuse | 16 |
| **Default** | 0.451 | 0.451 | 0.002 | 0.008 | MADRS-5 | 21 |
| **Visual** | 0.383 | 0.383 | 0.009 | 0.019 | MADRS-5 | 21 |
| **Dorsal Attention** | 0.299 | 0.299 | 0.046 | 0.046 | MADRS-10 | 27 |
| **Limbic** | 0.466 | -0.466 | 0.001 | 0.008 | MADRS-2 | 33 |
| **Somatomotor** | 0.299 | 0.299 | 0.046 | 0.046 | MADRS-2 | 33 |

***Table S29.*** *Significant correlations between PRS features and ROIs according to AAL atlas from the GMD map, ranked alphabetically.*

***Column definition:*** *PRS feature: name of the PRS feature used in the clinical+PRS model, refer to supplement S25 for the other column definitions.*

| **AAL ROI** | **r (abs)** | **r** | **P** | **P (fdr)** | **PRS feature** | **Feature weight rank** |
| --- | --- | --- | --- | --- | --- | --- |
| Angular_L | 0.365 | -0.365 | 0.047 | 0.049 | PRS_SZ_phi_1e.1 | 32 |
| Angular_R | 0.509 | 0.509 | 0.004 | 0.042 | PRS_SZ_phi_1e.5 | 34 |
| Angular_R | 0.473 | 0.473 | 0.008 | 0.042 | PRS_SZ_phi_1e.3 | 18 |
| Angular_R | 0.470 | 0.470 | 0.009 | 0.042 | PRS_SZ_phi_1e.6 | 35 |
| Angular_R | 0.469 | 0.469 | 0.009 | 0.042 | PRS_SZ_phi_1e.4 | 25 |
| Angular_R | 0.387 | 0.387 | 0.035 | 0.044 | PRS_SZ_phi_1e.1 | 32 |
| Angular_R | 0.377 | 0.377 | 0.040 | 0.047 | PRS_SZ_phi_1e.2 | 24 |
| Calcarine_R | 0.362 | 0.362 | 0.049 | 0.049 | PRS_SZ_phi_1e.3 | 18 |
| Cerebelum_3_R | 0.403 | 0.403 | 0.027 | 0.042 | PRS_SZ_phi_1e.1 | 32 |
| Cerebelum_6_L | 0.398 | -0.398 | 0.029 | 0.043 | PRS_SZ_phi_1e.1 | 32 |
| Cerebelum_6_R | 0.409 | 0.409 | 0.025 | 0.042 | PRS_SZ_phi_1e.2 | 24 |
| Cerebelum_6_R | 0.374 | 0.374 | 0.042 | 0.047 | PRS_SZ_phi_1e.3 | 18 |
| Cerebelum_Crus1_L | 0.401 | -0.401 | 0.028 | 0.042 | PRS_EA_phi_1e.4 | 3 |
| Cerebelum_Crus1_L | 0.401 | -0.401 | 0.028 | 0.042 | PRS_EA_phi_1e.2 | 14 |
| Cerebelum_Crus1_L | 0.384 | -0.384 | 0.036 | 0.044 | PRS_EA_phi_1e.3 | 8 |
| Cerebelum_Crus1_L | 0.384 | -0.384 | 0.036 | 0.044 | PRS_EA_phi_1e.1 | 20 |
| Cerebelum_Crus1_R | 0.404 | 0.404 | 0.027 | 0.042 | PRS_SZ_phi_1e.1 | 32 |
| Cerebelum_Crus2_R | 0.423 | 0.423 | 0.020 | 0.042 | PRS_EA_phi_1e.1 | 20 |
| Cerebelum_Crus2_R | 0.408 | 0.408 | 0.025 | 0.042 | PRS_EA_phi_1e.2 | 14 |
| Cerebelum_Crus2_R | 0.366 | 0.366 | 0.046 | 0.049 | PRS_EA_phi_1e.3 | 8 |
| Cingulum_Mid_L | 0.366 | -0.366 | 0.046 | 0.049 | PRS_EA_phi_1e.1 | 20 |
| Cingulum_Post_L | 0.462 | -0.462 | 0.010 | 0.042 | PRS_EA_phi_1e.3 | 8 |
| Cingulum_Post_L | 0.456 | -0.456 | 0.011 | 0.042 | PRS_EA_phi_1e.4 | 3 |
| Cingulum_Post_L | 0.431 | -0.431 | 0.017 | 0.042 | PRS_EA_phi_1e.6 | 10 |
| Cingulum_Post_L | 0.431 | -0.431 | 0.017 | 0.042 | PRS_EA_phi_1e.5 | 4 |
| Cingulum_Post_L | 0.407 | -0.407 | 0.025 | 0.042 | PRS_EA_phi_1e.2 | 14 |
| Cingulum_Post_L | 0.403 | -0.403 | 0.027 | 0.042 | PRS_EA_phi_1e.1 | 20 |
| Frontal_Inf_Orb_L | 0.435 | 0.435 | 0.016 | 0.042 | PRS_SZ_phi_1e.6 | 35 |
| Frontal_Inf_Orb_R | 0.403 | 0.403 | 0.027 | 0.042 | PRS_SZ_phi_1e.4 | 25 |
| Frontal_Inf_Orb_R | 0.384 | 0.384 | 0.036 | 0.044 | PRS_SZ_phi_1e.1 | 32 |
| Frontal_Inf_Orb_R | 0.380 | 0.380 | 0.038 | 0.046 | PRS_SZ_phi_1e.3 | 18 |
| Frontal_Inf_Orb_R | 0.372 | 0.372 | 0.043 | 0.048 | PRS_SZ_phi_1e.2 | 24 |
| Frontal_Med_Orb_R | 0.505 | -0.505 | 0.004 | 0.042 | PRS_EA_phi_1e.6 | 10 |
| Frontal_Med_Orb_R | 0.493 | -0.493 | 0.006 | 0.042 | PRS_EA_phi_1e.5 | 4 |
| Frontal_Med_Orb_R | 0.492 | -0.492 | 0.006 | 0.042 | PRS_EA_phi_1e.4 | 3 |
| Frontal_Med_Orb_R | 0.390 | -0.390 | 0.033 | 0.044 | PRS_EA_phi_1e.3 | 8 |
| Frontal_Med_Orb_R | 0.362 | -0.362 | 0.049 | 0.049 | PRS_EA_phi_1e.2 | 14 |
| Frontal_Mid_Orb_R | 0.457 | 0.457 | 0.011 | 0.042 | PRS_SZ_phi_1e.6 | 35 |
| Frontal_Mid_Orb_R | 0.442 | 0.442 | 0.015 | 0.042 | PRS_SZ_phi_1e.5 | 34 |
| Frontal_Mid_Orb_R | 0.407 | 0.407 | 0.026 | 0.042 | PRS_SZ_phi_1e.4 | 25 |
| Frontal_Sup_Orb_R | 0.571 | 0.571 | 0.001 | 0.042 | PRS_SZ_phi_1e.3 | 18 |
| Frontal_Sup_Orb_R | 0.569 | 0.569 | 0.001 | 0.042 | PRS_SZ_phi_1e.4 | 25 |
| Frontal_Sup_Orb_R | 0.520 | 0.520 | 0.003 | 0.042 | PRS_SZ_phi_1e.2 | 24 |
| Frontal_Sup_Orb_R | 0.476 | 0.476 | 0.008 | 0.042 | PRS_SZ_phi_1e.5 | 34 |
| Frontal_Sup_Orb_R | 0.438 | 0.438 | 0.015 | 0.042 | PRS_SZ_phi_1e.6 | 35 |
| Frontal_Sup_Orb_R | 0.403 | 0.403 | 0.027 | 0.042 | PRS_SZ_phi_1e.1 | 32 |
| Hippocampus_R | 0.444 | 0.444 | 0.014 | 0.042 | PRS_SZ_phi_1e.3 | 18 |
| Hippocampus_R | 0.377 | 0.377 | 0.040 | 0.047 | PRS_SZ_phi_1e.2 | 24 |
| Hippocampus_R | 0.364 | 0.364 | 0.048 | 0.049 | PRS_SZ_phi_1e.4 | 25 |
| Occipital_Inf_R | 0.510 | -0.510 | 0.004 | 0.042 | PRS_EA_phi_1e.5 | 4 |
| Occipital_Inf_R | 0.502 | -0.502 | 0.005 | 0.042 | PRS_EA_phi_1e.4 | 3 |
| Occipital_Inf_R | 0.471 | -0.471 | 0.009 | 0.042 | PRS_EA_phi_1e.3 | 8 |
| Occipital_Inf_R | 0.449 | -0.449 | 0.013 | 0.042 | PRS_EA_phi_1e.2 | 14 |
| Occipital_Inf_R | 0.438 | -0.438 | 0.016 | 0.042 | PRS_EA_phi_1e.6 | 10 |
| Occipital_Inf_R | 0.430 | -0.430 | 0.018 | 0.042 | PRS_EA_phi_1e.1 | 20 |
| Pallidum_L | 0.454 | -0.454 | 0.012 | 0.042 | PRS_EA_phi_1e.6 | 10 |
| Pallidum_L | 0.443 | -0.443 | 0.014 | 0.042 | PRS_EA_phi_1e.5 | 4 |
| Pallidum_L | 0.424 | -0.424 | 0.020 | 0.042 | PRS_EA_phi_1e.4 | 3 |
| Pallidum_L | 0.394 | -0.394 | 0.031 | 0.044 | PRS_EA_phi_1e.3 | 8 |
| Pallidum_L | 0.375 | -0.375 | 0.041 | 0.047 | PRS_SZ_phi_1e.6 | 35 |
| Pallidum_L | 0.365 | -0.365 | 0.047 | 0.049 | PRS_SZ_phi_1e.4 | 25 |
| ParaHippocampal_R | 0.449 | 0.449 | 0.013 | 0.042 | PRS_EA_phi_1e.2 | 14 |
| ParaHippocampal_R | 0.421 | 0.421 | 0.021 | 0.042 | PRS_EA_phi_1e.3 | 8 |
| ParaHippocampal_R | 0.417 | 0.417 | 0.022 | 0.042 | PRS_EA_phi_1e.1 | 20 |
| ParaHippocampal_R | 0.401 | 0.401 | 0.028 | 0.042 | PRS_EA_phi_1e.5 | 4 |
| Parietal_Inf_L | 0.415 | 0.415 | 0.022 | 0.042 | PRS_EA_phi_1e.3 | 8 |
| Parietal_Inf_L | 0.386 | 0.386 | 0.035 | 0.044 | PRS_EA_phi_1e.6 | 10 |
| Parietal_Sup_L | 0.470 | 0.470 | 0.009 | 0.042 | PRS_SZ_phi_1e.3 | 18 |
| Parietal_Sup_L | 0.440 | 0.440 | 0.015 | 0.042 | PRS_SZ_phi_1e.4 | 25 |
| Parietal_Sup_L | 0.364 | 0.364 | 0.048 | 0.049 | PRS_SZ_phi_1e.2 | 24 |
| Postcentral_R | 0.416 | -0.416 | 0.022 | 0.042 | PRS_EA_phi_1e.2 | 14 |
| Postcentral_R | 0.401 | -0.401 | 0.028 | 0.042 | PRS_EA_phi_1e.1 | 20 |
| Precentral_R | 0.470 | 0.470 | 0.009 | 0.042 | PRS_SZ_phi_1e.4 | 25 |
| Precentral_R | 0.425 | 0.425 | 0.019 | 0.042 | PRS_SZ_phi_1e.3 | 18 |
| Precentral_R | 0.407 | 0.407 | 0.026 | 0.042 | PRS_SZ_phi_1e.2 | 24 |
| Precentral_R | 0.398 | 0.398 | 0.029 | 0.043 | PRS_SZ_phi_1e.6 | 35 |
| Precentral_R | 0.391 | 0.391 | 0.033 | 0.044 | PRS_SZ_phi_1e.5 | 34 |
| Precuneus_L | 0.509 | -0.509 | 0.004 | 0.042 | PRS_EA_phi_1e.4 | 3 |
| Precuneus_L | 0.499 | -0.499 | 0.005 | 0.042 | PRS_EA_phi_1e.5 | 4 |
| Precuneus_L | 0.488 | -0.488 | 0.006 | 0.042 | PRS_EA_phi_1e.3 | 8 |
| Precuneus_L | 0.483 | -0.483 | 0.007 | 0.042 | PRS_EA_phi_1e.6 | 10 |
| Precuneus_L | 0.462 | -0.462 | 0.010 | 0.042 | PRS_EA_phi_1e.2 | 14 |
| Precuneus_L | 0.415 | -0.415 | 0.023 | 0.042 | PRS_EA_phi_1e.1 | 20 |
| Rectus_L | 0.387 | -0.387 | 0.034 | 0.044 | PRS_SZ_phi_1e.1 | 32 |
| Rectus_L | 0.384 | -0.384 | 0.036 | 0.044 | PRS_SZ_phi_1e.2 | 24 |
| Rectus_R | 0.363 | 0.363 | 0.049 | 0.049 | PRS_SZ_phi_1e.1 | 32 |
| Rolandic_Oper_L | 0.440 | 0.440 | 0.015 | 0.042 | PRS_SZ_phi_1e.3 | 18 |
| Rolandic_Oper_L | 0.422 | 0.422 | 0.020 | 0.042 | PRS_SZ_phi_1e.4 | 25 |
| Rolandic_Oper_L | 0.391 | 0.391 | 0.033 | 0.044 | PRS_SZ_phi_1e.2 | 24 |
| Rolandic_Oper_R | 0.433 | 0.433 | 0.017 | 0.042 | PRS_SZ_phi_1e.4 | 25 |
| Rolandic_Oper_R | 0.384 | 0.384 | 0.036 | 0.044 | PRS_SZ_phi_1e.3 | 18 |
| SupraMarginal_R | 0.432 | -0.432 | 0.017 | 0.042 | PRS_EA_phi_1e.6 | 10 |
| SupraMarginal_R | 0.387 | -0.387 | 0.035 | 0.044 | PRS_EA_phi_1e.5 | 4 |
| Vermis_3 | 0.375 | -0.375 | 0.041 | 0.047 | PRS_SZ_phi_1e.3 | 18 |
| Vermis_3 | 0.366 | -0.366 | 0.047 | 0.049 | PRS_SZ_phi_1e.1 | 32 |
| Vermis_7 | 0.443 | 0.443 | 0.014 | 0.042 | PRS_SZ_phi_1e.4 | 25 |
| Vermis_7 | 0.421 | 0.421 | 0.021 | 0.042 | PRS_SZ_phi_1e.3 | 18 |
| Vermis_7 | 0.391 | 0.391 | 0.033 | 0.044 | PRS_SZ_phi_1e.2 | 24 |

***Table S30.*** *Significant correlations between PRS features and ROIs according to AAL atlas from the GMD map summed by AAL brain regions, ranked by ROI count.*

***Column definition:*** *refer to supplement S26.*

| **AAL ROI** | **ROI count** | **Mean P (fdr)** |
| --- | --- | --- |
| **Frontal** | 19 | 0.020 |
| **Cerebellum + Vermis** | 17 | 0.027 |
| **Angular** | 7 | 0.046 |
| **Cingulum** | 7 | 0.018 |
| **Occipital** | 6 | 0.008 |
| **Pallidum** | 6 | 0.045 |
| **Precuneus** | 6 | 0.021 |
| **Parietal** | 5 | 0.029 |
| **Precentral** | 5 | 0.021 |
| **Hippocampus** | 3 | 0.046 |
| **Postcentral** | 2 | 0.042 |
| **SupraMarginal** | 2 | 0.043 |
| **Calcarine** | 1 | 0.049 |

***Table S31.*** *Significant correlations between PRS features and ROIs according to AAL atlas from the GMD map summed by PRS features, ranked by Feature weight rank.*

***Column definition:*** *PRS feature: name of the PRS feature used in the clinical+PRS model, refer to supplement S27 for the other column definitions.*

| **PRS feature** | **ROI count** | **Mean P (fdr)** | **Feature weight rank** |
| --- | --- | --- | --- |
| **PRS_EA_phi_1e.4** | 6 | 0.042 | 3 |
| **PRS_EA_phi_1e.5** | 7 | 0.042 | 4 |
| **PRS_EA_phi_1e.3** | 9 | 0.044 | 8 |
| **PRS_EA_phi_1e.6** | 7 | 0.042 | 10 |
| **PRS_EA_phi_1e.2** | 8 | 0.043 | 14 |
| **PRS_SZ_phi_1e.3** | 12 | 0.044 | 18 |
| **PRS_EA_phi_1e.1** | 8 | 0.043 | 20 |
| **PRS_SZ_phi_1e.2** | 10 | 0.045 | 24 |
| **PRS_SZ_phi_1e.4** | 11 | 0.043 | 25 |
| **PRS_SZ_phi_1e.1** | 10 | 0.045 | 32 |
| **PRS_SZ_phi_1e.5** | 4 | 0.043 | 34 |
| **PRS_SZ_phi_1e.6** | 6 | 0.043 | 35 |

***Table S32.*** *Significant correlations between PRS features and brain networks according to Yeo atlas from the GMD map, ranked by Feature weight rank.*

***Column definition:*** *Brain network: 7 brain networks defined by Yeo atlas, PRS feature: name of the PRS feature used in the clinical+PRS model, refer to supplement S25 for the other column definitions.*

| **Brain network** | **r (abs)** | **r** | **P** | **P (fdr)** | **PRS feature** | **Feature weight rank** |
| --- | --- | --- | --- | --- | --- | --- |
| **Frontalparietal** | 0.611 | 0.611 | 0.000 | 0.001 | PRS_SZ_phi_1e.3 | 18 |
| **Frontalparietal** | 0.545 | 0.545 | 0.002 | 0.005 | PRS_SZ_phi_1e.2 | 24 |
| **Frontalparietal** | 0.619 | 0.619 | 0.000 | 0.001 | PRS_SZ_phi_1e.4 | 25 |
| **Somatomotor** | 0.414 | 0.414 | 0.023 | 0.026 | PRS_SZ_phi_1e.4 | 25 |
| **Frontalparietal** | 0.421 | 0.421 | 0.021 | 0.026 | PRS_SZ_phi_1e.1 | 32 |
| **Frontalparietal** | 0.511 | 0.511 | 0.004 | 0.008 | PRS_SZ_phi_1e.5 | 34 |
| **Frontalparietal** | 0.472 | 0.472 | 0.009 | 0.014 | PRS_SZ_phi_1e.6 | 35 |
| **Somatomotor** | 0.375 | 0.375 | 0.041 | 0.041 | PRS_SZ_phi_1e.6 | 35 |

#### **C6. RESIS active and sham models post-hoc predicted treatment effects analyses**

We performed a set of post-hoc analyses to investigate the relationship between our models’ predictions, the patients’ treatment response rates and their PANSS-NS score changes observed at different follow-ups after patients completed treatment. First, we calculated the actual response rates in the predicted responder and non-responder strata as determined by our active models and compared these response rates against the treatment response rate observed in the non-stratified active/sham rTMS samples using χ^2^ tests. Second, we conducted linear regression analyses to calculate the correlations (R²) between the predicted rTMS response likelihood and the observed PANSS-NS score reduction after 21 days of active treatment. Lastly, we calculated the effect sizes (Cohen’s d) of the differences between PANSS-NS scores at 21-days timepoint and the baseline scores using the following formula:

Cohen's *d* = (*M*PANSS-NS 21 days - *M*PANSS-NS baseline) ⁄ *SD*pooled,

where *SD*pooled = √((*SD*PANSS-NS baseline2 + *SD*PANSS-NS 21 days2) ⁄ 2).

Figure S33, table S34 and S35 show that the strata induced by the active rTMS models’ predictions were characterized by higher treatment response rates compared to the non-stratified patients, which responded to the active or sham intervention in 46.7% of cases. The treatment response rates in the active models’ predicted responder strata were consistently higher compared non-stratified patients, with the highest response rate (90.9%) observed in the predicted response stratum of the sequential model. Accordingly, the non-response rates in the predicted responder strata were consistently lower than in non-stratified sample with the lowest non-response rate (4.3%) found in the sequential model’s predicted responder stratum (table S34). When comparing treatment response rates in the predicted responders strata to the response rates of the non-stratified patient cohort, the sMRI model (p=.026), sMRI+Clinical model (p=.014), all modalities stacker (p=.012) and sequential model (p=.004) showed significantly higher rates (table S35).

***Figure S33.*** *Line plot of the response and non-response rates of all active models and the non-stratified active /sham treated patient groups. The treatment response rate of the non-stratified active rTMS patient group was identical to the non-stratified sham-treated patient group. Models with significantly different treatment response rates compared to all active rTMS patients were marked with *. Refer to Table S35 for the χ^2^ tests’ P values.*

*
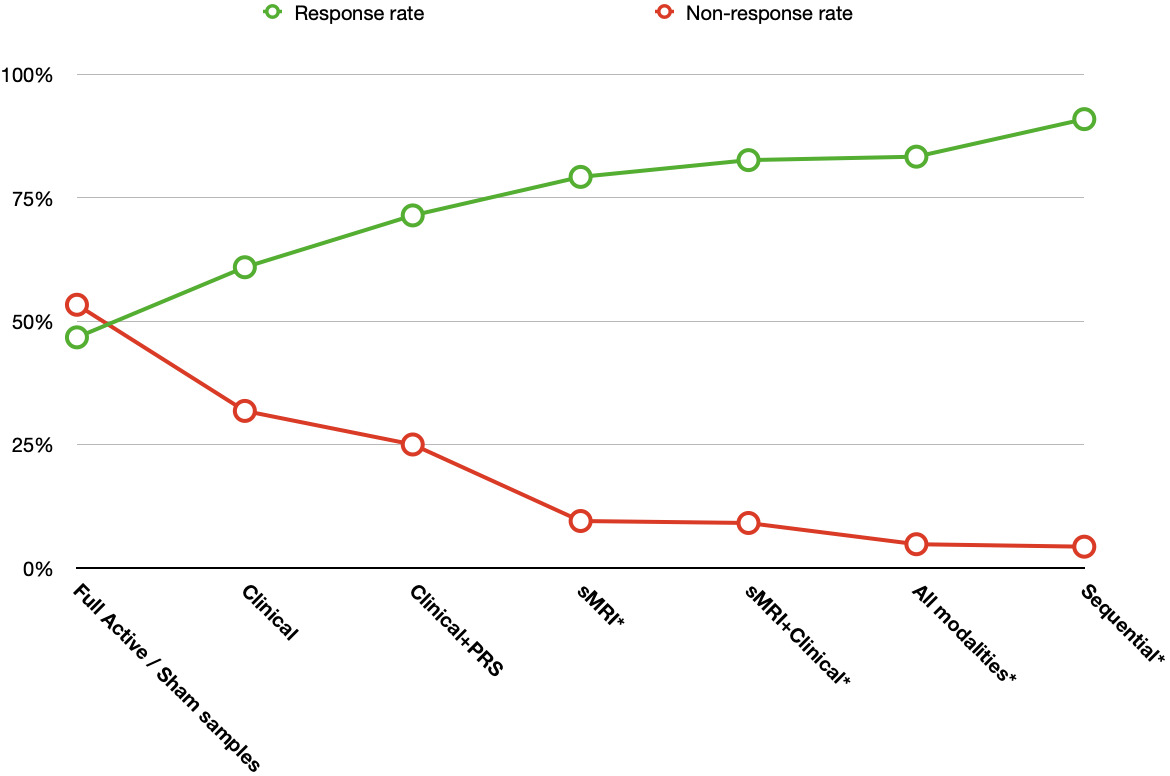
*

Figure S36 and table S37 show that the active-group model predictions were significantly correlated with the PANSS-NS score reductions after 21 days of treatment, ranging from R² of 0.20 to 0.41, except for the clinical-only model. We observed large effect sizes (Cohen’s d >0.80) in patients with PANSS-NS response predictions and medium (Cohen’s d <0.50) to small (Cohen’s d <0.20) effect sizes in patients with predicted PANSS-NS non-responses in all active-group models. Furthermore, significant differences between baseline and 21 days PANSS-NS scores were found in the active-rTMS models’ predicted responder strata (t-p (Resp) fdr < .05), while no significant PANSS-NS differences were found in patients with non-response predictions. These results showed that all active models successfully stratified responders and non-responders in the active treatment group (Figure S36, left). On the contrary, we did not find significant linear correlations between predicted response likelihoods and PANSS-NS score reductions at completion of the treatment in models trained on sham group (Figure S36, right). These results further support the specificity of our proposed models for active rTMS and illustrate the benefits of the RCT setup in developing clinically relevant machine learning models.

***Table S34.*** *Treatment response counts and response rates of all models trained on the active rTMS sample as well as the of the non-stratified active/sham-treated patients.*

***Column definitions:*** *Model: model name, Group: the predicted subgroup name, N Predicted Responders: number of patients with a treatment response at 21-days follow-up timepoint, N Predicted Non-responders: number of patients without a treatment response at 21-days follow-up timepoint, Response rate (%): ratio of N Predicted Responders to N Predicted Non-responders.*

| **Model** | **Stratum in the active rTMS group** | **N Predicted Responders** | **N Predicted Non-responders** | **Response rate (%)** |
| --- | --- | --- | --- | --- |
| Active Clinical | predicted response | 14 | 9 | 60.9 |
| Active Clinical | predicted non-response | 7 | 15 | 31.8 |
| Active Clinical+PRS | predicted response | 15 | 6 | 71.4 |
| Active Clinical+PRS | predicted non-response | 6 | 18 | 25 |
| Active sMRI | predicted response | 19 | 5 | 79.2 |
| Active sMRI | predicted non-response | 2 | 19 | 9.5 |
| Active sMRI+Clinical | predicted response | 19 | 4 | 82.6 |
| Active sMRI+Clinical | predicted non-response | 2 | 20 | 9.1 |
| Active All modalities | predicted response | 20 | 4 | 83.3 |
| Active All modalities | predicted non-response | 1 | 20 | 4.8 |
| Active Sequential | predicted response | 20 | 2 | 90.9 |
| Active Sequential | predicted non-response | 1 | 22 | 4.3 |
| Full active / sham | response | 21 | 24 | 46.7 |

***Table 35.*** *Comparisons of treatment response rates of responder and non-responder strata as predicted by the active rTMS models against the non-stratified active/sham-treated patients.*

***Column definitions:*** *Model: model name, Comparison: description of the treatment responses used in the respective χ^2^ comparison, χ^2^: test statistic, p: P values obtained from χ^2^ tests, p (fdr): FDR-corrected P values. All significant P values were marked with *.*

| **Model** | **Comparison** | **χ^2^** | **p** | **p (fdr)** |
| --- | --- | --- | --- | --- |
| Active Clinical | predicted response vs full active / sham | 0.73 | 0.394 | 0.394 |
| Active Clinical | predicted non-response vs full active / sham | 0.8 | 0.372 | 0.393 |
| Active Clinical | predicted response vs predicted non-response | 2.74 | 0.098 | 0.126 |
| Active Clinical+PRS | predicted response vs full active / sham | 2.61 | 0.106 | 0.127 |
| Active Clinical+PRS | predicted non-response vs full active / sham | 2.24 | 0.134 | 0.151 |
| Active Clinical+PRS | predicted response vs predicted non-response | 7.92 | 0.005 | 0.011* |
| Active sMRI | predicted response vs full active / sham | 5.52 | 0.019 | 0.026* |
| Active sMRI | predicted non-response vs full active / sham | 7.14 | 0.008 | 0.012* |
| Active sMRI | predicted response vs predicted non-response | 19.12 | 0.000 | 0.000* |
| Active sMRI+Clinical | predicted response vs full active / sham | 6.7 | 0.010 | 0.014* |
| Active sMRI+Clinical | predicted non-response vs full active / sham | 7.66 | 0.006 | 0.011* |
| Active sMRI+Clinical | predicted response vs predicted non-response | 21.55 | 0.000 | 0.000* |
| Active All modalities | predicted response vs full active / sham | 7.27 | 0.007 | 0.012* |
| Active All modalities | predicted non-response vs full active / sham | 9.51 | 0.002 | 0.005* |
| Active All modalities | predicted response vs predicted non-response | 24.71 | 0.000 | 0.000* |
| Active Sequential | predicted response vs full active / sham | 10.39 | 0.001 | 0.004* |
| Active Sequential | predicted non-response vs full active / sham | 10.6 | 0.001 | 0.004* |
| Active Sequential | predicted response vs predicted non-response | 30.46 | 0.000 | 0.000* |

***Figure S36.*** ***Left:*** *Scatter plot comparison of linear correlations between patients’ predicted likelihood of non-response to rTMS treatment from all models trained on active-treated group and PANSS-NS score reduction from baseline to 21 days after rTMS treatment.* ***Right:*** *Scatter plot comparison of linear correlations between patients’ predicted likelihood of non-response to rTMS treatment from all models trained on sham-treated group and PANSS-NS score reduction from baseline to 21 days after rTMS treatment.*


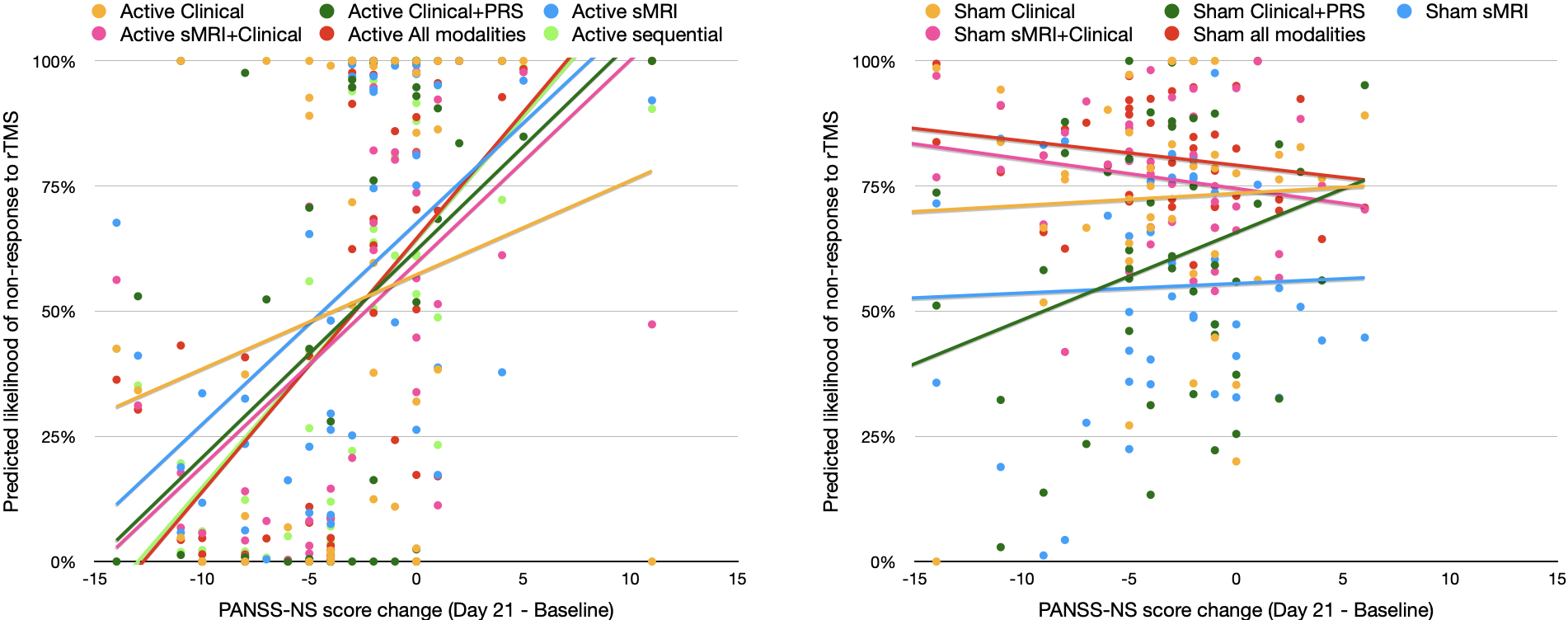


***Table S37.*** *Comparison of linear correlations and effect sizes between patients’ predicted likelihood of non-response to rTMS treatment from all models trained on both active and sham-treated groups and PANSS-NS score reduction from baseline to 21 days after initiation of rTMS treatment.*

***Column definitions:*** *R²: R-squared value of the linear regression analysis between the model’s predicted likelihood of non-response and PANSS-NS score reduction, R² P (fdr): FDR-corrected p-value of the R-squared value from the linear regression, Cohen’s d (Resp): effect size measure of the predicted response patients’ PANSS-NS score reduction, t (Resp): statistics from t-test between the baseline and 21 days PANSS-NS scores of the predicted responders, t-p (Resp) fdr: FDR-corrected p-value from t-test between the baseline and 21 days PANSS-NS scores of the predicted responders, Cohen’s d (Non-Resp): effect size measure of the predicted non-responders’ PANSS-NS score reduction, t (Non-Resp): statistics from t-test between the baseline and 21 days PANSS-NS scores of the predicted non-responders, t-p (Non-Resp) fdr: FDR-corrected p-value from t-test between the baseline and 21 days PANSS-NS scores of the predicted non-responders.*

| **Active models** | **R²** | **R² p**  **fdr** | **Cohen's d (Resp)** | **t**  **(Resp)** | **t-p**  **(Resp) fdr** | **Cohen's d (Non-Resp)** | **t**  **(Non-Resp)** | **t-p**  **(Non-Resp) fdr** |
| --- | --- | --- | --- | --- | --- | --- | --- | --- |
| Observed label | 0.58 | <.001 | 1.68 | 9.90 | <.001 | 0.03 | -0.27 | .825 |
| Clinical | 0.04 | <.001 | 0.83 | 3.82 | <.001 | 0.37 | 2.54 | .133 |
| Clinical+PRS | 0.20 | <.001 | 1.15 | 6.44 | <.001 | 0.26 | 1.42 | .397 |
| sMRI | 0.27 | <.001 | 1.05 | 5.97 | <.001 | 0.17 | 0.97 | .597 |
| sMRI+Clinical | 0.26 | <.001 | 0.99 | 4.84 | <.001 | 0.23 | 1.57 | .397 |
| All modalities | 0.38 | <.001 | 1.27 | 7.66 | <.001 | 0.03 | -0.20 | .852 |
| Sequential | 0.41 | <.001 | 1.28 | 7.60 | <.001 | 0.03 | 0.19 | .852 |
| **Sham**  **models** | **R²** | **R² p**  **fdr** | **Cohen's d (Resp)** | **t**  **(Resp)** | **t-p**  **(Resp) fdr** | **Cohen's d (Non-Resp)** | **t**  **(Non-Resp)** | **t-p**  **(Non-Resp) fdr** |
| Observed label | 0.55 | <.001 | 1.65 | 8.72 | <.001 | 0.13 | 1.08 | .293 |
| Clinical | 0.00 | .787 | 0.99 | 1.67 | .157 | 0.81 | 5.03 | <.001 |
| Clinical+PRS | 0.09 | .121 | 1.19 | 3.40 | .006 | 0.67 | 4.11 | <.001 |
| sMRI | 0.00 | .787 | 0.68 | 3.20 | .006 | 0.95 | 4.28 | <.001 |
| sMRI+Clinical | 0.05 | .242 | NA | NA | NA | 0.78 | 5.15 | <.001 |
| All modalities | 0.05 | .242 | NA | NA | NA | 0.80 | 5.35 | <.001 |

Figure S38 and table S39 show the predictive performance of the active rTMS models in a longitudinal setting. Specifically, the results showed the linear correlations between patients’ predicted likelihood of non-response to rTMS treatment from sMRI, all modalities stacker and sequential models trained on active-treated group and PANSS-NS score reduction from baseline to different follow-up time periods. We observed that the predicted response likelihoods from the active rTMS models were significantly correlated with PANSS-NS score reductions between baseline and 21 days, 28 days, and 45 days after initiation of treatment. The respective R² values decreased across all models with increased follow-up duration, from the highest correlations observed at the 21-days follow-up to the lowest correlations at the 105-days follow-up. These results combined with the longitudinal increase of Cohen’s D in the predicted non-responsive patients may indicate that the treatment effect of the rTMS diminishes over time.

***Figure S38.*** *Scatter plot comparison of linear correlations between patients’ predicted likelihood of non-response to rTMS treatment from sMRI, all modalities stacker and sequential models trained on active-treated group and PANSS-NS score reduction from baseline to different follow-up time periods after rTMS treatment.* ***Top-Left:*** *PANSS-NS score reduction from baseline to 21 days after rTMS treatment.* ***Top-Right:*** *PANSS-NS score reduction from baseline to 28 days after rTMS treatment.* ***Bottom-Left:*** *PANSS-NS score reduction from baseline to 45 days after rTMS treatment.* ***Bottom-Right:*** *PANSS-NS score reduction from baseline to 105 days after rTMS treatment.*

*
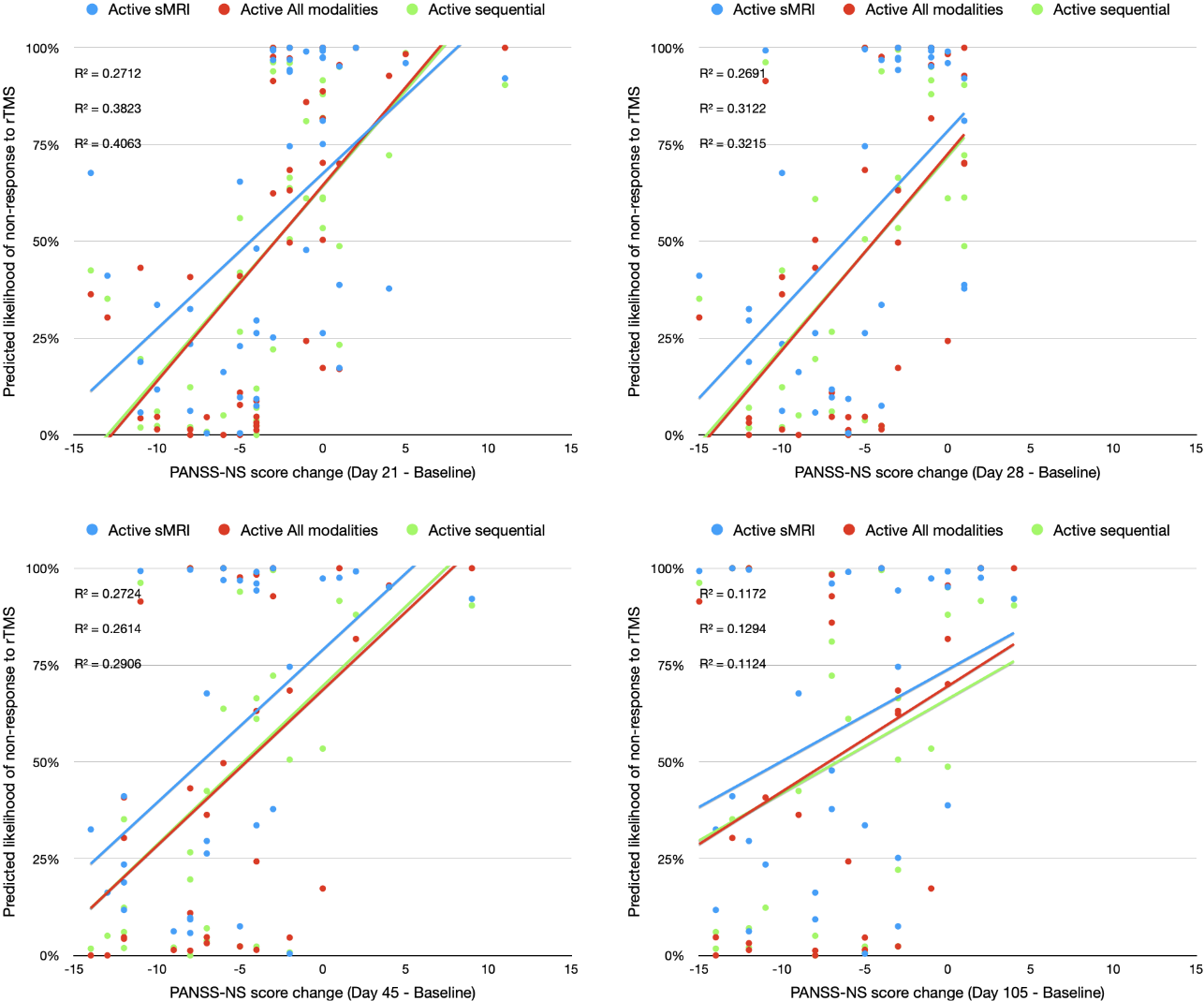
*

***Table S39.*** *Comparison of linear correlations and effect sizes between patients’ predicted likelihood of non-response to rTMS treatment from sMRI, all modalities stacker and sequential models trained on active-treated group and PANSS-NS score reduction from baseline to different follow-up time periods after rTMS treatment.*

***Column definitions:*** *refer to supplement S34.*

| **Active Models** | **Follow-up timepoint** | **R²** | **R² P (FDR)** | **Cohen’s d (Resp)** | **Cohen’s d (Non-Resp)** |
| --- | --- | --- | --- | --- | --- |
| sMRI | 21 days | 0.271 | < .001* | 1.05 | 0.17 |
| All modalities | 21 days | 0.382 | < .0001* | 1.27 | -0.03 |
| Sequential | 21 days | 0.496 | < .0001* | 1.28 | 0.03 |
| sMRI | 28 days | 0.269 | .001* | 1.82 | 0.54 |
| All modalities | 28 days | 0.312 | .0006* | 1.7 | 0.47 |
| Sequential | 28 days | 0.321 | .0006* | 1.72 | 0.52 |
| sMRI | 45 days | 0.272 | .003* | 2.09 | 0.47 |
| All modalities | 45 days | 0.261 | .003* | 1.68 | 0.39 |
| Sequential | 45 days | 0.291 | .003* | 1.76 | 0.45 |
| sMRI | 105 days | 0.117 | .07 | 1.96 | 0.73 |
| All modalities | 105 days | 0.129 | .07 | 1.77 | 0.76 |
| Sequential | 105 days | 0.112 | .07 | 1.68 | 0.81 |

**References**

Behdenna, A., Haziza, J., Azencott, C.-A., & Nordor, A. (2020). *PyComBat, a Python tool for batch effects correction in high-throughput molecular data using empirical Bayes methods* (p. 2020.03.17.995431). bioRxiv. https://doi.org/10.1101/2020.03.17.995431

Gómez-Verdejo, V., Parrado-Hernández, E., Tohka, J., & Alzheimer’s Disease Neuroimaging Initiative. (2019). Sign-Consistency Based Variable Importance for Machine Learning in Brain Imaging. *Neuroinformatics*, *17*(4), 593–609. https://doi.org/10.1007/s12021-019-9415-3

He, H., Bai, Y., Garcia, E. A., & Li, S. (2008). ADASYN: Adaptive synthetic sampling approach for imbalanced learning. *2008 IEEE International Joint Conference on Neural Networks (IEEE World Congress on Computational Intelligence)*, 1322–1328. https://doi.org/10.1109/IJCNN.2008.4633969

Johnson, W. E., Li, C., & Rabinovic, A. (2007). Adjusting batch effects in microarray expression data using empirical Bayes methods. *Biostatistics*, *8*(1), 118–127. https://doi.org/10.1093/biostatistics/kxj037

Koutsouleris, N., Dwyer, D. B., Degenhardt, F., Maj, C., Urquijo-Castro, M. F., Sanfelici, R., Popovic, D., Oeztuerk, O., Haas, S. S., Weiske, J., Ruef, A., Kambeitz-Ilankovic, L., Antonucci, L. A., Neufang, S., Schmidt-Kraepelin, C., Ruhrmann, S., Penzel, N., Kambeitz, J., Haidl, T. K., … PRONIA Consortium. (2021). Multimodal Machine Learning Workflows for Prediction of Psychosis in Patients With Clinical High-Risk Syndromes and Recent-Onset Depression. *JAMA Psychiatry*, *78*(2), 195–209. https://doi.org/10.1001/jamapsychiatry.2020.3604

Koutsouleris, N., Wobrock, T., Guse, B., Langguth, B., Landgrebe, M., Eichhammer, P., Frank, E., Cordes, J., Wölwer, W., Musso, F., Winterer, G., Gaebel, W., Hajak, G., Ohmann, C., Verde, P. E., Rietschel, M., Ahmed, R., Honer, W. G., Dwyer, D., … Hasan, A. (2018). Predicting Response to Repetitive Transcranial Magnetic Stimulation in Patients With Schizophrenia Using Structural Magnetic Resonance Imaging: A Multisite Machine Learning Analysis. *Schizophr Bull*, *44*(5), 1021–1034. https://doi.org/10.1093/schbul/sbx114

Krishnan, A., Williams, L. J., McIntosh, A. R., & Abdi, H. (2011). Partial Least Squares (PLS) methods for neuroimaging: A tutorial and review. *NeuroImage*, *56*(2), 455–475. https://doi.org/10.1016/j.neuroimage.2010.07.034

Rorden, C., & Brett, M. (2000). Stereotaxic display of brain lesions. *Behavioural Neurology*, *12*(4), 191–200. https://doi.org/10.1155/2000/421719

Thomas Yeo, B. T., Krienen, F. M., Sepulcre, J., Sabuncu, M. R., Lashkari, D., Hollinshead, M., Roffman, J. L., Smoller, J. W., Zöllei, L., Polimeni, J. R., Fischl, B., Liu, H., & Buckner, R. L. (2011). The organization of the human cerebral cortex estimated by intrinsic functional connectivity. *Journal of Neurophysiology*, *106*(3), 1125–1165. https://doi.org/10.1152/jn.00338.2011

[Wobrock, T. *et al.* Left prefrontal high-frequency repetitive transcranial magnetic stimulation for the treatment of schizophrenia with predominant negative symptoms: a sham-controlled, randomized multicenter trial. *Biol Psychiatry* **77**, 979–88 (2015).](https://www.zotero.org/google-docs/?cj9jxo)
